# Recent Progresses in Constructing the Highly Efficient Ni Based Catalysts With Advanced Low-Temperature Activity Toward CO_2_ Methanation

**DOI:** 10.3389/fchem.2020.00269

**Published:** 2020-04-28

**Authors:** Chufei Lv, Leilei Xu, Mindong Chen, Yan Cui, Xueying Wen, Yaping Li, Cai-e Wu, Bo Yang, Zhichao Miao, Xun Hu, Qinghui Shou

**Affiliations:** ^1^Jiangsu Key Laboratory of Atmospheric Environment Monitoring and Pollution Control, Collaborative Innovation Center of the Atmospheric Environment and Equipment Technology, School of Environmental Science and Engineering, Nanjing University of Information Science & Technology, Nanjing, China; ^2^College of Light Industry and Food Engineering, Nanjing Forestry University, Nanjing, China; ^3^School of Chemistry and Chemical Engineering, Shandong University of Technology, Zibo, China; ^4^School of Material Science and Engineering, University of Jinan, Jinan, China; ^5^CAS Key Laboratory of Bio-Based Materials, Qingdao Institute of Bioenergy and Bioprocess Technology (QIBEBT), Chinese Academy of Sciences (CAS), Qingdao, China

**Keywords:** carbon dioxide, methanation, Ni-based catalyst, low-temperature catalytic activity, recent progresses

## Abstract

With the development and prosperity of the global economy, the emission of carbon dioxide (CO_2_) has become an increasing concern. Its greenhouse effect will cause serious environmental problems, such as the global warming and climate change. Therefore, the worldwide scientists have devoted great efforts to control CO_2_ emissions through various strategies, such as capture, resource utilization, sequestration, etc. Among these, the catalytic conversion of CO_2_ to methane is considered as one of the most efficient routes for resource utilization of CO_2_ owing to the mild reaction conditions and simple reaction device. Pioneer thermodynamic studies have revealed that low reaction temperature is beneficial to the high catalytic activity and CH_4_ selectivity. However, the low temperature will be adverse to the enhancement of the reaction rate due to kinetic barrier for the activation of CO_2_. Therefore, the invention of highly efficient catalysts with promising low temperature activities toward CO_2_ methanation reaction is the key solution. The Ni based catalysts have been widely investigated as the catalysts toward CO_2_ methanation due to their low cost and excellent catalytic performances. However, the Ni based catalysts usually perform poor low-temperature activities and stabilities. Therefore, the development of highly efficient Ni based catalysts with excellent low-temperature catalytic performances has become the research focus as well as challenge in this field. Therefore, we summarized the recent research progresses of constructing highly efficient Ni based catalysts toward CO_2_ methanation in this review. Specifically, the strategies on how to enhance the catalytic performances of the Ni based catalysts have been carefully reviewed, which include various influencing factors, such as catalytic supports, catalytic auxiliaries and dopants, the fabrication methods, reaction conditions, etc. Finally, the future development trend of the Ni based catalysts is also prospected, which will be helpful to the design and fabrication of the Ni catalysts with high efficiency toward CO_2_ methanation process.

## Introduction

In the context of the rapid development of global economy, the energy crisis, and environmental pollution has been becoming more and more serious. The consumption of a large number of fossil fuels has led to a sharp rise in CO_2_ emissions. In 2017, the total CO_2_ emissions reached 41 billion tons, attracting the increasing worldwide attention. Therefore, how to control and reduce the amount of CO_2_ in the atmosphere has become an urgent issue (Gac et al., 2018; Zhang G. et al., 2018; Li W. et al., 2019). The strategies for CO_2_ emission reduction mainly cover capture-storage as well as capture-conversion. However, gas leakage is a crucial problem with capture and storage technologies, and it is also difficult to choose suitable storage sites. Chemical conversion is considered as the most convenient and effective method (Bian et al., 2018). In 1902, Sabatier proposed the process of methanation of CO_2_ (Zhang G. et al., 2018). The process is a reaction which could convert the sustainable hydrogen energy from solar energy and biomass into methane with high CO_2_ conversion rate and high CH_4_ selectivity. The results of thermodynamic calculation show that higher pressure and lower temperature are more suitable for methanation process (Su et al., 2016; Champon et al., 2019). Compared to reactions with C_2+_ hydrocarbons as the products, CO_2_ methanation has relatively lower requirements on reaction temperature and pressure and has much broader application prospects, such as replacing natural gas production with syngas. The CO_2_ methanation reaction achieves the goal of reducing environmental pollution by converting CO_2_ into value-added CH_4_, which is also a kind of clean energy with high calorific value and can alleviate the problem of energy shortage. Therefore, the CO_2_ methanation process can be considered as the one of the most effective routes toward CO_2_ emission control at present (Solis-Garcia et al., 2017; Gnanakumar et al., 2019).

As regarding the CO_2_ methanation reaction, the most concerning factors are the reaction rate and chemical equilibrium. From the perspective of thermodynamics, the CO_2_ methanation reaction will generate a large amount of heat release (−165 kJ/mol) due to the exothermic feature of this process. Therefore, when the reaction temperature is higher than 627°C, it is supposed to obtain lower CO_2_ conversion and CH_4_ selectivity. However, the change in Gibbs free energy will be >0 according to thermodynamic calculation and the reverse reaction will take place, namely, CH_4_ reacts with H_2_O to form CO_2_ (Hu et al., 2019). From the perspective of dynamics, CO_2_ methanation is an eight-electron involved process with obvious kinetic barrier. This process demands high temperature to overcome the kinetic barrier to reduce the stable CO_2_ (+4) with strong C = O bond into CH_4_ (−4). Low temperature will reduce the reaction rate based on the dynamic theory (Alarcón et al., 2019). In order to obtain high theoretical CO_2_ conversion and CH_4_ selectivity, the whole reaction process is desirable to carry out at low temperature (Jiang et al., 2019; Ou et al., 2019). Therefore, it is of great necessity to employ highly efficient catalyst to achieve advanced low-temperature catalytic activity.

As well-known, the catalyst are mainly composed of active center, catalytic support, and catalytic dopant, among which the active center is considered as the key component of the catalyst. It was reported that most of the VIII group metals, such as Ru, Rh, Pd, Ni, Co, and Fe, could act as the active centers toward CO_2_ methanation process. Among these metals, Pd, Rh, and Ru are all precious metals. Compared with other non-precious metal catalysts, the precious metal based catalysts have better low-temperature catalytic activities, and higher methane selectivity. However, they are not suitable for large-scale promotion and application due to the limitations of high price and rare resources (Panagiotopoulou, 2017; Chai et al., 2019). Although Co-based catalysts have excellent low-temperature activity and stability, yet they usually have low methane selectivity (Li et al., 2018a; Liu H. et al., 2018). As for the Fe-based catalysts, they may tend to accumulate carbon over the catalyst surface, easily form liquid hydrocarbons, and are not suitable for low reaction temperatures (Kirchner et al., 2018). Consequently, Ni-based catalysts are widely investigated as the catalysts toward CO_2_ methanation due to their excellent catalytic performances and low price, though their low-temperature activities and anti-sintering properties are not excellent enough. Therefore, the development of Ni-based catalysts with outstanding low-temperature activity and endurable stability has been considered as an important Research Topic in this field. For the Ni-based catalysts, their catalytic activities are not as high as those of noble metal catalysts. Besides, the heat release and low reaction temperature will also lead to the formation of carbonyl nickel, and then Ni sintering and deactivation will occur (Veselovskaya et al., 2018). In order to address these drawbacks, the researchers have devoted great efforts to study the influence of different supports and auxiliaries on Ni-based catalysts. Bacariza et al. (2018a) reported that SBA-15 and MCM-41 could prevent the sintering process due to their large pore sizes and interesting textural properties. Valinejad Moghaddam et al. (2018) found that all the investigated additives except Cu could promote the CO_2_ conversion of the catalysts modified by Fe, Co, Zr, La, and Cu. Ni-Fe/Al_2_O_3_ catalyst showed the best catalytic performance because the addition of Fe changed the physical properties of the catalyst and increased the number of surface exposed active centers. In addition, some researchers have devoted themselves to exploring the effect of preparation conditions on Ni-based catalysts. It was reported that the calcination temperature could affect the structure and activity of the catalyst (Haynes et al., 2019). In general, the catalytic activity of Ni-based catalysts can be improved by tuning various influencing factors of the catalyst.

**Figure 1:**
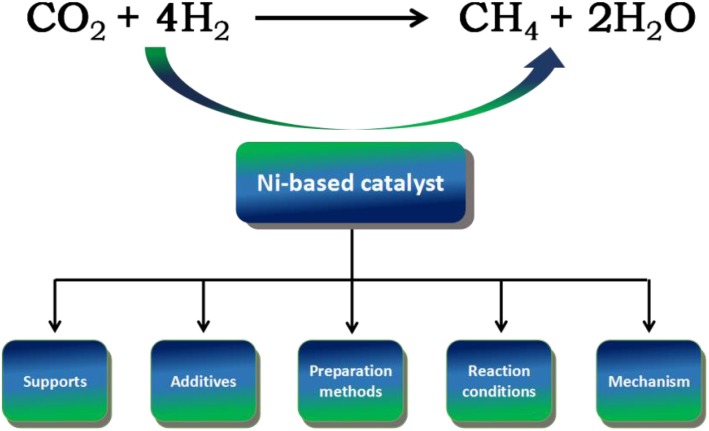
The main outline of this review.

In recent years, many scholars have summarized the development of catalysts from different aspects. For example, Stangeland et al. (2017a) studied the CO_2_ methanation from the perspective of the reaction conditions of catalysts. Aziz and Jalil (2015) discussed recent developments in heterogeneous catalysts with emphases on their physicochemical properties, catalytic activities, and reaction mechanism. Ni-based catalysts are commonly used in CO_2_ methanation due to its low cost and high activity. However, the review on this topic is somewhat incomplete and the perspective of the relevant literature summary of recent research progress is not sufficient. Therefore, it is of great significance to summarize the recent development of Ni-based catalysts with excellent low-temperature activity. The main outline of this review is displayed in Figure 1. Specifically, the subject of this review is focused on the influencing parameters of the catalyst design on the low-temperature catalytic performance of Ni-based catalysts. Furthermore, the reaction mechanism of CO_2_ methanation over different Ni-based catalysts is also summarized. Finally, the future development trend of Ni-based catalysts is also prospected in this review.

## Types of NI-based Catalysts

Structural property, nickel dispersion, and strong metal-support interaction are important index factors of Ni-based catalysts, which affect the low-temperature catalytic activity and sintering-resistant performance of Ni-based catalysts. Therefore, it is important to design and develop novel catalysts with suitable porosity, high nickel dispersion, and strong metal-support interaction (Gac et al., 2018). High nickel dispersion is usually achieved by employing microporous or mesoporous supports with excellent structural property and doping various modifiers, such as Co, Zr, Sm, etc. (Li et al., 2020). Besides, the support can also inhibit or even avoid the serious sintering of metal nanoparticles by enhancing the strong metal-support interaction and affect the activity of CO_2_ methanation by promoting the dissociation and adsorption of CO_2_ (Ye et al., 2019). The catalytic performances of the representative catalysts are summarized in Table 1 based on overall discussion of Ni-based catalysts.

**Table 1:** The summaries of the structural properties and catalytic performances of the Ni-based catalysts toward CO_2_ methanation.

**Catalysts**	**Pore diameter (nm)**	**X_**CO2**_ (%)**	**S_**CH4**_ (%)**	**T (**°**C)**	**Stability test, conversion (%)**	**References**
15 wt% Ni/TiO_2_	5.8	80.1	95.0	340	Decreased by 16.5% after 81 h.	Liu et al., 2013
10 wt% Ni/ZrO_2_	5.4	74.2	70.8	—	—	Jia et al., 2019
Ni30/Al_2_O_3_	5.4	70.7	95.0	—	—	Gac et al., 2019
15Ni/CeO_2_0.6ZrO_2_0.4	9.1	71.0	86.0	300	Decreased from 83% to 70% after 42 h.	Pastor-Pérez et al., 2018
25Ni/Ce0.75Zr0.25	—	85.0	—	300	Stable within 30 h.	Atzori et al., 2019
30Ni/Al_2_O_3_.0.5SiO_2_	4.8	82.0	98.0	350	Stable within 30 h.	Moghaddam et al., 2018
20Ni/Al_2_O_3_-ZrO_2_-1.0	3.3	76.0	100.0	300	Stable within 100 h.	Lin et al., 2019
15Ni/OMA	8.2	87.0	98.0	400	Stable within 150 h.	Aljishi et al., 2018
5%Ni/MSN	3.4	—	99.9	300	Stable within 200 h.	Aziz et al., 2014a
12Ni/CNT	3.7	61.1	96.6	350	Decreased by 18% after 100 h.	Wang W. et al., 2016
5%Ni/fibrous SBA-15	7.0	98.9	99.6	400	Stable within 120 h.	Bukhari et al., 2019
NiO@SiO_2_	11.6	78.0	98.0	—	—	Li et al., 2014
Ni0.8Mg0.2O@SiO_2_	15.5	87.0	99.0	300	Stable within 100 h.	Li et al., 2014
OMA-10Ni8Ca	8.4	80.0	98.5	400	Stable within 50 h.	Xu et al., 2017a
15Ni3Co/CeO_2_0.6ZrO_2_0.4	5.4	83.0	94.0	300	Decreased by 13% after 42 h.	Pastor-Pérez et al., 2018
2Co8Ni/OMA	9.5	79.0	97.5	400	Stable within 50 h.	Xu et al., 2018
Ni-Mn/γ-Al_2_O_3_	5.1	90.0	—	270	Stable within 50 h.	Le et al., 2019a
12Ni3Fe/Al_2_O_3_	5.2	84.1	100	420	Stable within 150 h.	Li et al., 2018b
MA-10Ni3La	11.1	78.0	98.3	400	Stable within 50 h.	Xu et al., 2017c
12Ni4.5Ce/CNT	3.7	83.8	99.8	350	Decreased by 1.3% after 100 h.	Wang W. et al., 2016
13Ni-2CeO_2_/Al_2_O_3_	12.5	85.0	99.0	350	Decreased by 2.0% after 120 h.	Liu H. et al., 2012
Ni(10 wt%)-Pt(0.5 wt%) /γ-Al_2_O_3_	—	83.4	97	250	Decreased by 2.5% after 6 h.	Mihet and Lazar, 2018
Ni(10 wt%)-Pd(0.5 wt%) /γ-Al_2_O_3_	—	90.6	97	250	Decreased by 3.4% after 6 h.	Mihet and Lazar, 2018

### Ni-Based Catalysts With Different Supports

As the skeleton of catalyst, the supports with large specific surface areas and large pore volumes can accommodate the Ni active centers in highly dispersed state (Bacariza et al., 2018a). The promotion of the redox property of electron transfer between the support and the metal active center will promise the increase of electron density in the metal. These electrons can enhance the coupling between nickel and carbon, thereby facilitating the C = O bond-breaking and the formation of CH_4_ (Liu W. et al., 2018). Besides, the physicochemical properties of different supports will affect the crystal size and surface properties of Ni as well as the reducibility and catalytic properties of catalysts. The hierarchical pore structure of the support could also promote the mass transfer of the feed stock on the catalyst surface (Pizzolitto et al., 2019). Meanwhile, the dispersion of the Ni species over the catalyst surface is also greatly affected by the structure of the support (Bian and Kawi, 2017). Furthermore, the chemisorption and activation of the CO_2_ over the support with strong alkalinity could be intensified and the activated CO_2_ will react quickly due to low kinetic barrier, which in turn makes the catalyst equipped with anti-coke deposition performance. Therefore, the catalytic support is closely related to the catalytic performance of the catalyst (Rönsch et al., 2016).

#### Single Type Catalytic Support

The single type catalytic supports with different morphologies are usually used as the supports for Ni based catalysts toward CO_2_ methanation, such as Al_2_O_3_, TiO_2_, SiO_2_, ZrO_2_, etc. (Ferreira and Branco, 2019). For example, TiO_2_, well-known as an N-type semiconductor with good thermal stability, can easily form the strong metal-support interaction with the active component Ni, so that CO_2_ can easily adsorb on the catalyst surface (Liu et al., 2013; Zhou R. et al., 2016). It was reported that the CO_2_ conversion over Ni/TiO_2_ catalyst was 96% at the low temperature (260°C) (Liu et al., 2013). ZrO_2_ is an amphoteric compound with good stability, abundant oxygen vacancies, and it can remain active under high temperature conditions (Jia et al., 2019; Tan et al., 2019). Besides, ZrO_2_ usually has three types of monoclinic (m-ZrO_2_), tetragonal (t-ZrO_2_), and cubic structure (Guilera et al., 2019). In terms of reaction mechanism, the catalytic activity of the catalyst is mainly related to the relative content of m-ZrO_2_ because m-ZrO_2_ usually has more oxygen vacancies, which are beneficial to the adsorption of oxygen-containing substances (Zhao et al., 2016a; Zhang X. et al., 2019). According to the report by Romero-Sáez et al. (2018), ZrO_2_ could not activate and decompose H_2_, but also could activate CO_2_ molecules to generate CO. Therefore, H_2_ molecule was dissociated on the surface of Ni and the CO_2_ molecule was activated on the surface of ZrO_2_ (Ocampo et al., 2011). Therefore, the interaction between hydrogen atoms and activated CO_2_ molecules could be promoted, and the selectivity of CH_4_ and reaction rate could be improved by increasing the range of the Ni-ZrO_2_ interface (Garbarino et al., 2019). CeO_2_ has good stability, strong CO_2_ adsorption performance and outstanding oxygen storage capacity, which promise the high low-temperature activity over its supported Ni based catalysts (Tada et al., 2012; Löfberg et al., 2017; Ratchahat et al., 2018; Yan et al., 2019). Specifically, the redox cycle between Ce^3+^/Ce^4+^ ion pairs on the surface of CeO_2_ can form the oxygen vacancy, which can promote the adsorption and activation of CO_2_ to generate CO during the reaction (Tada et al., 2012; Löfberg et al., 2017; Ratchahat et al., 2018; Yan et al., 2019). Al_2_O_3_ is a porous support with low cost and large specific surface area, promising the homogenous dispersion of Ni over the catalyst surface (Akbari et al., 2018; Ray et al., 2018). Besides, Al_2_O_3_ and Ni could form the NiAl_2_O_4_ structure with strong Ni-O chemical bond that hinders the reduction of Ni^2+^. Thus, the metallic Ni particles over the catalyst surface are small in crystalline size, which can inhibit the carbon deposition (Ahn et al., 2019).

**Figure 2:**
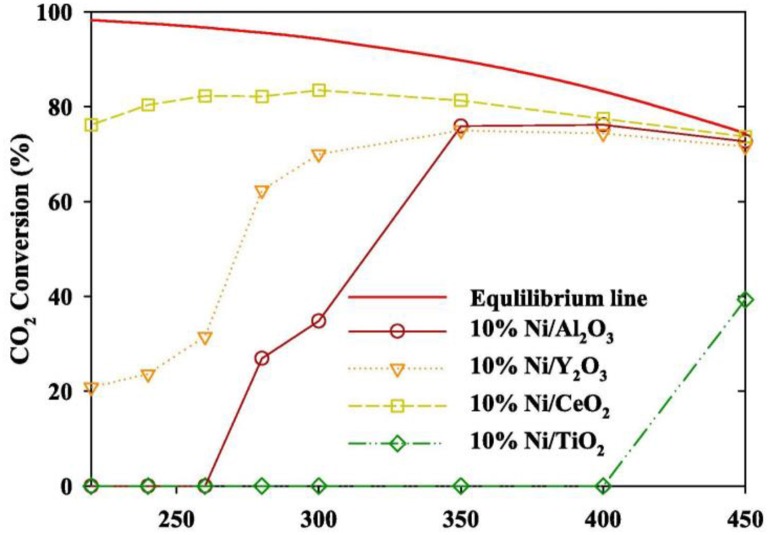
Evaluation of the CO_2_ methanation of a Ni catalyst containing different supports (inlet gas CO_2_: N_2_: H_2_ = 1: 1: 4, SV 14,400 h^−1^, catalyst loading 0.5 g). Reproduced from Ahn et al. (2019) with permission from Elsevier and Copyright Clearance Center.

In order to realize the high-efficient conversion of CO_2_ to CH_4_ at low temperature, the influences of different supports on catalysts are also discussed. Martínez et al. (2018) compared the effects of Ni-based catalysts supported over ZrO_2_, SiO_2_, and MgAl_2_O_4_ on the hydrogenation of CO_2_ to CH_4_. The CO_2_ conversion of all Ni-based catalysts was positively correlated with the reaction temperature. 20% Ni/ZrO_2_ catalyst exhibited the best catalytic activity and long-term stability. Specifically, the majority presence of tetragonal zirconia as well as the strong Ni-ZrO_2_ interaction were responsible for the high catalytic performance of the Ni/ZrO_2_ catalysts (Li et al., 2018b). Ahn et al. (2019) found that Ni/CeO_2_ catalysts exhibited the best stability and highest selectivity in Ni-based catalysts supported by TiO_2_, Al_2_O_3_, Y_2_O_3_, and CeO_2_, respectively. Figure 2 showed the CO_2_ conversion of Ni-based catalysts based on different supports and Ni/CeO_2_ had the highest CO_2_ conversion. Besides, it was found that high activation temperature was required for alumina supported catalysts because alumina-supported nickel catalysts were less reductive than CeO_2_ and ZrO_2_ (Gac et al., 2019). Therefore, CeO_2_ and ZrO_2_ are more suitable for carbon dioxide methanation under low temperature conditions.

#### Composite Catalytic Support

The composite catalytic supports for the Ni based catalysts for CO_2_ methanation mainly include CeO_2_-ZrO_2_, ZrO_2_-Al_2_O_3_, Al_2_O_3_-SiO_2_, etc. They can combine the characteristics of different supports together to better exert the catalytic performance of the catalyst than single support. In addition, the synergy between the composite supports would further affect the catalytic performance of CO_2_ methanation (Wang Y. et al., 2018; Champon et al., 2019).

**Figure 3:**
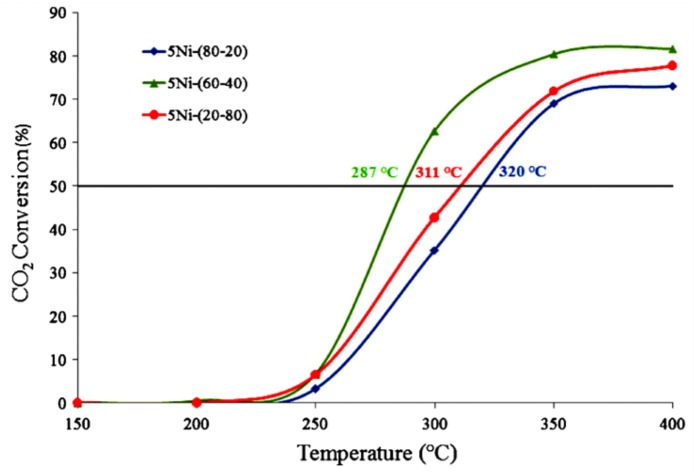
CO_2_ conversion vs. temperature over 5Ni–CZ catalytic systems. Reproduced from Ocampo et al. (2011) with permission from Elsevier and Copyright Clearance Center.

For the CeO_2_-ZrO_2_ support, the crystalline lattices of CeO_2_ and ZrO_2_ could infiltrate into each other in atomic level and form the solid solution. The incorporation of ZrO_2_ in the CeO_2_ causes defects in the structure, which can promote the activation of CO_2_ during the process of methanation (Rezaei and Alavi, 2019). It was reported that the catalytic performance of the Ni/CeO_2_-ZrO_2_ (CZ) catalyst was affected by the composition of the Ce/Zr ratio (Ocampo et al., 2011). As shown in Figure 3, the CO_2_ conversion over 5Ni/CZ (60–40) catalyst was higher than other catalysts. Specifically, the addition of ZrO_2_ increased the oxygen mobility in the lattice of cerium oxide and promoted the formation of vacancies, which in turn affected the consumption of H_2_ and the activation of CO_2_ rapidly (Bacani et al., 2017; Pastor-Pérez et al., 2018; Atzori et al., 2019). Iglesias et al. (2019) also found that Ni/CeO_2_-ZrO_2_ catalyst had higher oxygen vacancy rate and higher Ni utilization rate than Ni/CeO_2_. Therefore, the improvement of catalyst performance was mainly attributed to their high oxygen storage capacity, excellent oxygen vacancy, and high Ni dispersion.

As regard to the Al_2_O_3_-SiO_2_ support, its large specific surface area can expose much more accessible Ni active centers to the gaseous feedstock than the single counterparts. Besides, the interaction between Al_2_O_3_ and NiO is enhanced by the formation of strong chemical bonds, which can effectively hinder the accumulation of Ni on the catalyst surface. However, Al_2_O_3_ will react with NiO species to form NiAl_2_O_4_ when calcined at high temperature, which makes the reduction of the Ni species very difficult (Xu Y. et al., 2019). The presence of SiO_2_ can weaken the interaction between Ni and Al_2_O_3_ by inhibiting the phase transformation of Al_2_O_3_ from ɤ to α and hinder the serous accumulation of Ni species via obtaining the optimal co-dissociation energy. As a result, its catalytic performance under low temperature conditions can be greatly improved (Cai et al., 2014; Charisiou et al., 2019). Moghaddam et al. (2018) studied the catalytic performance of Ni-based catalysts supported by Al_2_O_3_-SiO_2_. Compared with the single support, the dispersion of the Ni active center over the Ni/Al_2_O_3_-SiO_2_ catalyst was better due to the larger surface area and bigger pore volume of the composite support, which made the hydrogenation of CO_2_ to methane more easier. Thus, this catalyst performed the best performance (82.38% CO_2_ conversion and 98.19% CH_4_ selectivity at 350°C) among all the catalysts.

**Figure 4:**
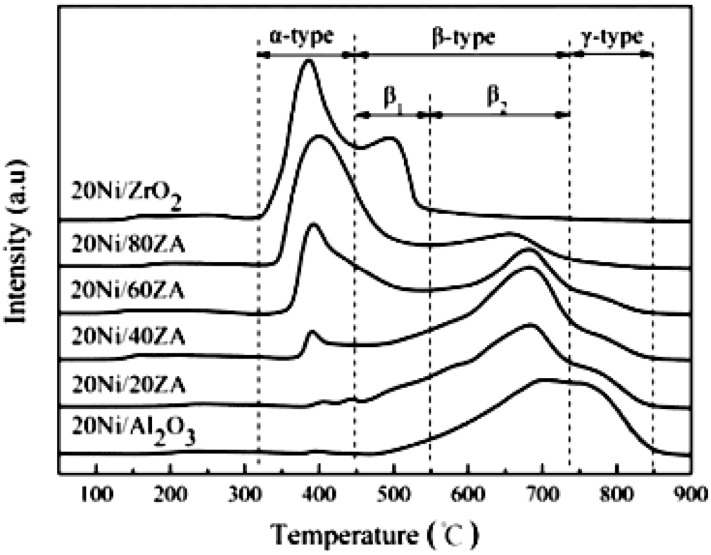
TPR profiles of catalysts with different ZrO_2_contents. Reproduced from Guo et al. (2014) with permission from Elsevier and Copyright Clearance Center.

**Figure 5:**
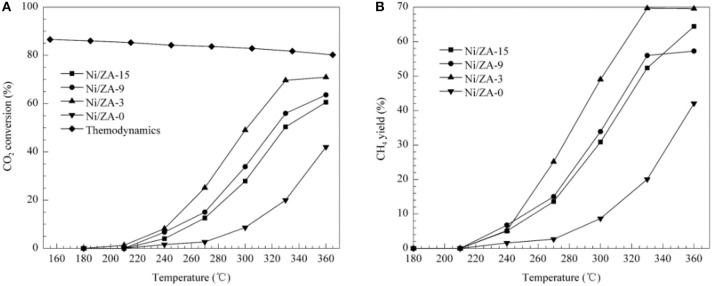
**(A)** CO_2_ conversion and **(B)** CH_4_ selectivity vs. temperature for the Ni/ZA-x (x = 0, 3, 9, 15) catalysts. Reaction conditions: 101.3 kPa, GHSV = 8,100 mL/(h·g_cat_), H_2_/CO_2_ molar ratio = 3.5. Reproduced from Cai et al. (2011) with permission from Elsevier and Copyright Clearance Center.

ZrO_2_-Al_2_O_3_ has excellently sintering-resistant and thermally stable properties (Cai et al., 2011). As well-known, Al_2_O_3_ suffers the disadvantage of poor thermal stability in the CO_2_ methanation reaction while ZrO_2_ can easily form stable interaction with NiO, which is beneficial to the improvement of stability toward CO_2_ methanation (Peymani et al., 2017; Jia et al., 2019). Therefore, the application of mesoporous ZrO_2_-Al_2_O_3_ composite support with unique structural properties and chemical properties is a novel strategy to prepare high efficient catalyst (Guo et al., 2014). As shown in Figure 4, the β-type peak of 20Ni/20ZrO_2_-Al_2_O_3_(ZA) was obviously higher than that of 20Ni/Al_2_O_3_. When the ZrO_2_ content of the support was <40%, the weakening effect of ZrO_2_ on the NiO-Al_2_O_3_ interaction was more obvious, and the reduction was easier to proceed with the increase of ZrO_2_ content (Guo et al., 2014; Liu et al., 2014). This phenomenon could be owing to the interaction between NiO and Al_2_O_3_ weakened by the presence of ZrO_2_ in ZrO_2_-Al_2_O_3_ support. As a result, the formation of NiAl_2_O_4_ was effectively inhibited and the Ni particles with smaller particle size were thus obtained. Therefore, the catalyst had excellent catalytic effect in CO_2_ methanation. In addition, more active metallic Ni^0^ active centers could be obtained under milder reduction conditions due to the lower content of the NiAl_2_O_4_. Therefore, the catalytic activity of the Ni/ZrO_2_-Al_2_O_3_ catalyst was higher than that of Ni/Al_2_O_3_ (Abate et al., 2016; Zhan et al., 2018). Cai et al. (2011) also found that the CO_2_ conversion over Ni/ZrO_2_ catalyst was much lower than that of Ni/ZrO_2_-Al_2_O_3_ as shown in Figure 5. The CO_2_ chemisorption and conversion had been intensified owing to the presence of the oxygen vacancies over the ZrO_2_-Al_2_O_3_ composite support, which was in favor of the hinderance of the carbon deposition and the improvement of the catalytic stability. Besides, the number of the oxygen vacancies and metallic Ni active centers over the catalyst surface gradually increased with the increase of the Zr loading, which could significantly improve the low-temperature catalytic activity and CH_4_ selectivity of the catalyst according to Lin et al. (2019).

#### Novel Support

The novel supports, such as mesoporous materials (Zhang G. et al., 2019), molecular sieves (Quindimil et al., 2018), nanotubes (László et al., 2016), grapheme (Hu et al., 2019), MOFs (Ghanbari et al., 2019), and ZIFs (Zhao et al., 2019), are commonly used. Compared with the traditional support, the novel supports mainly possess the following two aspects of advantages. Firstly, the novel supports usually possesses excellent textural structures, such as specific surface area, pore volume, pore diameter, etc. Take the zeolite as an example, it is a kind of microporous materials with high thermal stability, large surface area, and strong chemisorption for CO_2_, which are conducive to the improvement of catalytic activity and stability by providing more accessible active centers (Luengnaruemitchai and Kaengsilalai, 2008; Quindimil et al., 2018). Secondly, the novel support can endow the metallic active center in high dispersion state. For example, the graphene oxide can modify the electronic structure of Ni on the catalyst surface and reduce the dissociation energy of H_2_ and CO_2_ (He et al., 2016). Ma et al. (2019) prepared Ni-SiO_2_/GO-Ni-foam catalyst by intercalation method. Compared with the Ni-SiO_2_/Ni-foam catalyst, Ni-SiO_2_/GO-Ni-foam with larger surface area had stronger CO_2_ adsorption capacity and good anti-sintering ability. Besides, graphene oxide could enhance the interaction between Ni and SiO_2_ and the Ni-SiO_2_/GO-Ni-foam catalyst could form a large amount of Ni silicate, promising high dispersion of Ni.

**Figure 6:**
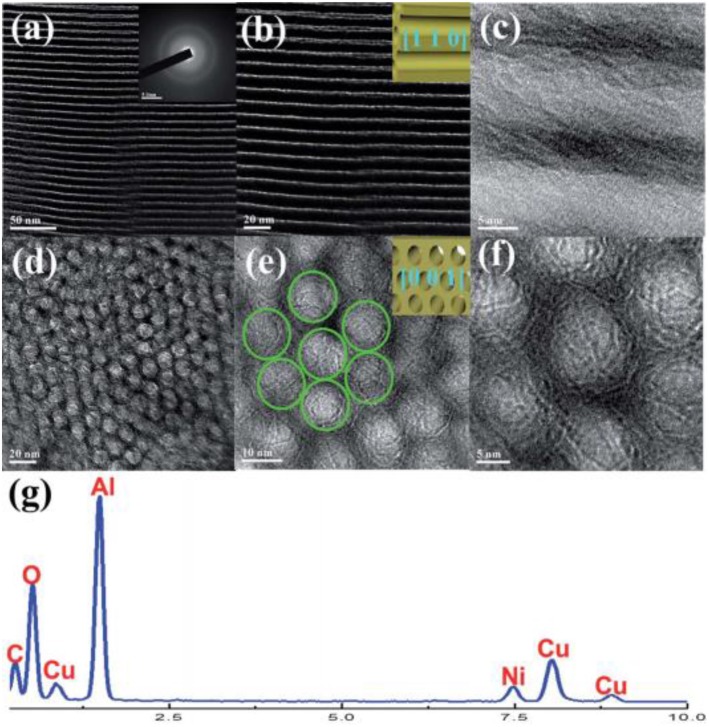
TEM **(a–e)**, SAED **(f)**, and EDS **(g)** measurements of the OMA−10Ni catalyst. Reproduced from Xu et al. (2016) with permission from Royal Society of Chemistry.

**Figure 7:**
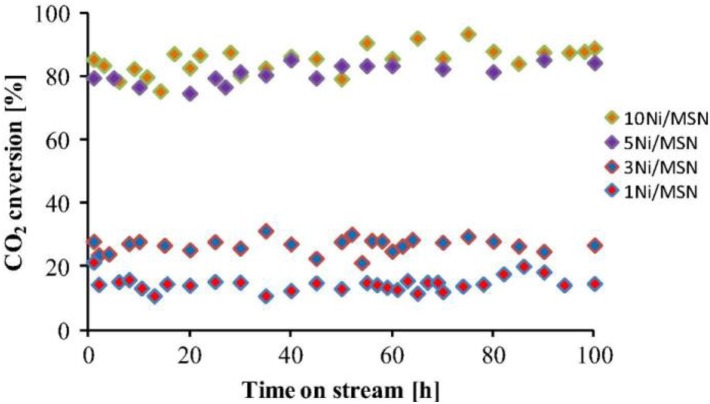
Stability test of all catalysts. Reaction temperature = 623 K, H_2_/CO_2_ = 4/1, GHSV = 50,000 mL ${\text{g}}_{\text{cat}}^{-1}$ h^−1^. Reproduced from Aziz et al. (2015) with permission from Elsevier and Copyright Clearance Center.

Mesoporous materials are considered as the ideal catalytic supports with novel structure. The mesoporous structure could not only confine the metal Ni to a fixed space, but also provide a large surface area for the high dispersion of the metallic active centers. Thereby, the stability of the catalyst could be greatly improved (Liu Q. et al., 2015). Recently, mesoporous alumina and mesoporous silica materials have been widely investigated as the catalytic supports toward different catalytic reactions. Shen et al. (2011) reported that mesoporous materials were excellent supports for preventing metal particle from sintering. Xu et al. (2016) found that ordered mesoporous NiO-Al_2_O_3_ (MA) exhibited higher catalytic activity and better stability than non-mesoporous materials(NPA) and Ni/ɤ-Al_2_O_3_ due to its outstanding structural property as shown in Figure 6. Specifically, the ordered mesoporous catalyst with uniform pore size could promise the facile access to the exposed Ni active sites for the gaseous feedstock, which would be beneficial to the diffusion of the reactant gases and accelerating the reaction speed. Besides, Xu et al. (2017a) synthesized Co/Ca modified ordered mesoporous Ni based catalysts by the evaporation-induced self-assembly (EISA) strategy to further improve the low-temperature catalytic activity of the catalyst. The alkaline earth and rare earth dopants are used to intensify the chemisorption and activation of CO_2_ (Li B. et al., 2019). Xu et al. (2018) found that the ordered mesoporous Al_2_O_3_ prepared by this method had uniform porous structure and outstanding thermal stability. The framework of MA could stabilize the particle size of Ni particles via the confinement effect of the mesoporous channel and strong metal-support interaction, which could effectively hinder the sintering of the Ni particle at high reaction temperatures (Xu L. et al., 2012). In addition, MA's unique structure was conducive to the mass transfer of both the gaseous reactants and products owing to outstanding structural properties (Liu Q. et al., 2016). Aljishi et al. (2018) found that the physical properties of ordered mesoporous nickel alumina catalyst (Ni/OMA) were in favor of the uniform dispersion and the formation of more accessible Ni reaction centers, thus promoting the CO_2_ methanation reaction. It was reported that the CO_2_ conversion and CH_4_ selectivity of the catalyst Ni/OMA was higher than those of traditional catalysts in the range of 300–500°C (Liu et al., 2018a). Meanwhile, the hydrothermal stability of Ni/OMA was good and the fiber structure of the catalyst was successfully retained after the catalytic reaction. As for mesoporous silica, it has a high surface area, on which the high dispersion of Ni active centers could be achieved (Li et al., 2018c). Besides, it was reported that the high activity of Ni/MSN may be due to the existence of both interparticle porosity and the high basicity of the catalysts (Aziz et al., 2014a). Specifically, the basic sites could promote the chemisorption of CO_2_ on the catalyst surface, thus increasing the reaction rate. The interparticle pores could facilitate the mass transport of the reactants and product molecules during the catalytic reaction and thus allowing higher CO_2_ conversion to be obtained (Wang et al., 2006). In addition, Aziz et al. (2015) found that the CO_2_ conversion rate over Ni/MSN catalyst did not decrease significantly during the stability test in Figure 7, showing excellent coke resistance and good stability within 200 h time on stream. The phenomenon may be ascribed to the strong metal-support interaction between the MSN and Ni metal, which could endow the catalyst with high carbon deposition resistance (Xiang et al., 2016).

**Figure 8:**
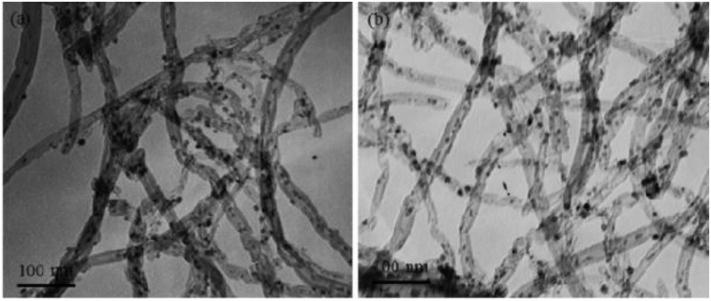
TEM images of 12Ni4.5Ce/CNT catalyst before **(a)** and after **(b)** stability test. Reproduced from Wang W. et al. (2016) with permission from Elsevier and Copyright Clearance Center.

In recent years, carbon nanotubes have been widely used as the support of the catalysts due to their excellent properties (Andersen et al., 2015). It has a unique structure and can enhance the dispersion of metallic Ni. Meanwhile, the carbon nanotubes can improve the reduction performance and catalytic activity of the catalyst because the binding effect of the CNTs has a confinement effect on the particle size of the Ni particles, which can hinder the migration and sintering of the active component Ni (Xiong et al., 2013). In addition, active centers with strong adsorption and conductivity exist in the interior and surface of carbon nanotubes, which can promote the electron transfer of active substances (Li J. et al., 2018). Wang W. et al. (2016) compared Ce-doped Ni-based catalysts with Al_2_O_3_ and carbon nanotubes as supports. Compared with the catalyst with Al_2_O_3_ as the support, the carbon nanotube-supported catalyst had a larger specific surface area. Therefore, as shown in Figure 8, the average particle size of the metallic Ni particles was about 3–5 nm before the CO_2_ methanation reaction. The particle size of metallic Ni was also not substantially changed after the stability test. This indicated that the CNT support can effectively limit the Ni particle size in the tubular structure below the nanometer scale.

**Figure 9:**
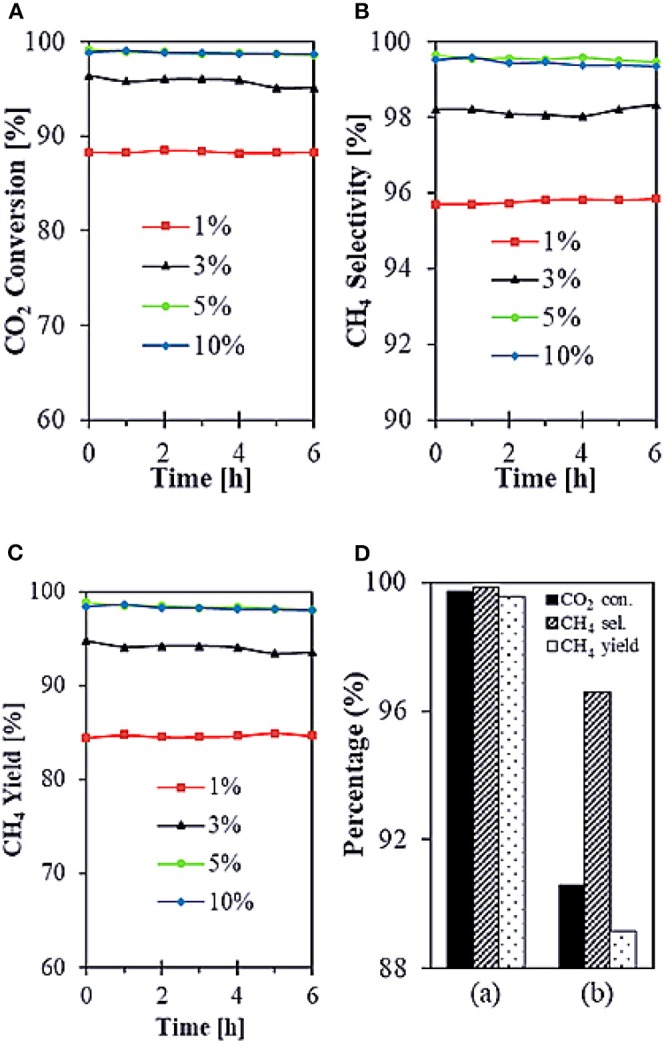
**(A–C)** Catalytic performances of all different Ni loadings onto F-SBA-15 support (1, 3, 5, and 10%) toward CO_2_ methanation. Reaction conditions: T = 673 K, GHSV = 24,900 mL g^−1^ h^−1^, stoichiometric H_2_/CO_2_ = 4/1, time-on-stream = 6 h. **(D)** Comparison performance of (a) 5%Ni/F-SBA-15 toward CO_2_ methanation with (b) 5%Ni/SBA-15. Reproduced from Bukhari et al. (2019) with permission from Elsevier and Copyright Clearance Center.

SiO_2_ is widely considered as a common support for Ni-based catalysts. However, Ni/SiO_2_ catalysts are prone to generate carbon deposits during the reaction and thus cause inactivation (Wang F. et al., 2018). Therefore, many scholars have studied the structure of SiO_2_ in order to solve this problem. Zhang L. et al. (2019) reported that core-shell structured Ni@SiO_2_ catalysts fabricated by microemulsion method could achieve strong metal-support interaction, which was beneficial to preserve the small size of Ni nanoparticles during the reaction. Therefore, Ni@SiO_2_ catalysts had excellent catalytic performance. In addition, Bukhari et al. (2019) synthesized the Ni-based catalyst supported on fibrous SBA-15 (F-SBA-15) by impregnation method. As shown in Figure 9, 5%Ni/F-SBA-15 exhibited 98.9% CO_2_ conversion and 99.6% CH_4_ selectivity at 673 K for 6 h time-on-stream because the fibrous morphology and dendrimeric structure of F-SBA-15 were in favor of Ni dispersion.

### Nickel-Based Catalyst Doped With Different Additives

Ni-based catalysts always have some inherent drawbacks, such as poor low-temperature activity and serious thermal sintering at high temperature. The incorporation of catalytic dopants has been considered as an effective strategies to improve these drawbacks (Alrafei et al., 2019). It was reported that the modified catalyst with adequate additive usually had a higher CO_2_ conversion than the corresponding reference catalyst (Li Y. et al., 2019). Although the catalytic additive itself has no evidently catalytic activity, it can significantly promote the catalytic performances by regulating the dispersion of the metallic active center and surface properties. Specifically, the dispersion of the active component can be improved by changing the structure of the catalyst and the electronic property and/or alkalinity of the catalyst surface could be modified due to the incorporation of the catalytic additives. As a result, the reaction barrier in terms of the apparent activation energy can be greatly lowered and as a result, the low-temperature catalytic activity could be greatly promoted (Wang Y. et al., 2018). For the bimetal and/or multifetal based catalyst, the synergistic effect between Ni with the incorporated metal also improves the dissociation and desorption capacity of the catalyst for H_2_ and CO_2_, thereby enhancing the methanation reactivity (Liu X. et al., 2012). For an instance, Wang X. et al. (2018) found that Ce-doped catalysts not only had good catalytic stability, but also had outstanding adaptability to different reaction conditions because the synergy between the metals Ce and Ni promoted the activation of CO_2_ at lower temperatures. In addition, the incorporated dopants, such as Zr, Co, La, Mg, can change the reaction pathways of CO_2_ methanation and reaction intermediates. As a result, the production of CO can be effectively avoided owing to the effective hinderance of the side reaction. Liang et al. (2019a)found that all the Cr, Mn, Fe, Co, La, and Ce modified catalysts were provided with multiple oxygen vacancies, promising higher catalytic activities than Ni-Al_2_O_3_ catalysts by the enhancement of the chemisorption and activation of CO_2_ adsorption. The reason for promoted catalytic performance was that these modified catalysts experienced the formate-type intermediates, which could be converted to CH_4_ more rapidly.

#### Single Additive

The type of single additives for Ni based catalysts can be mainly classified into alkaline earth metal oxides, transition metals, rare earth metal oxides, and noble metals. They can improve catalytic performance by adjusting the structural parameters of the catalyst, such as specific surface area and pore size, the electronic effect, and surface basicity, which would influence the interaction between the catalyst and the CO_2_ molecule (Su et al., 2016).

##### Alkaline earth metal oxides

The addition of alkaline earth metal oxides, such as MgO, CaO, SrO, and BaO, have been widely considered as a series of structural additives, which can obviously increase the surface basicity of the catalyst. Besides, Sr-modified catalysts can also generate the oxygen vacancies and prevent electron pairing. Ba can inhibit CO formation by inhibiting the reverse water-gas shift reaction (Liang et al., 2019b).

**Figure 10:**
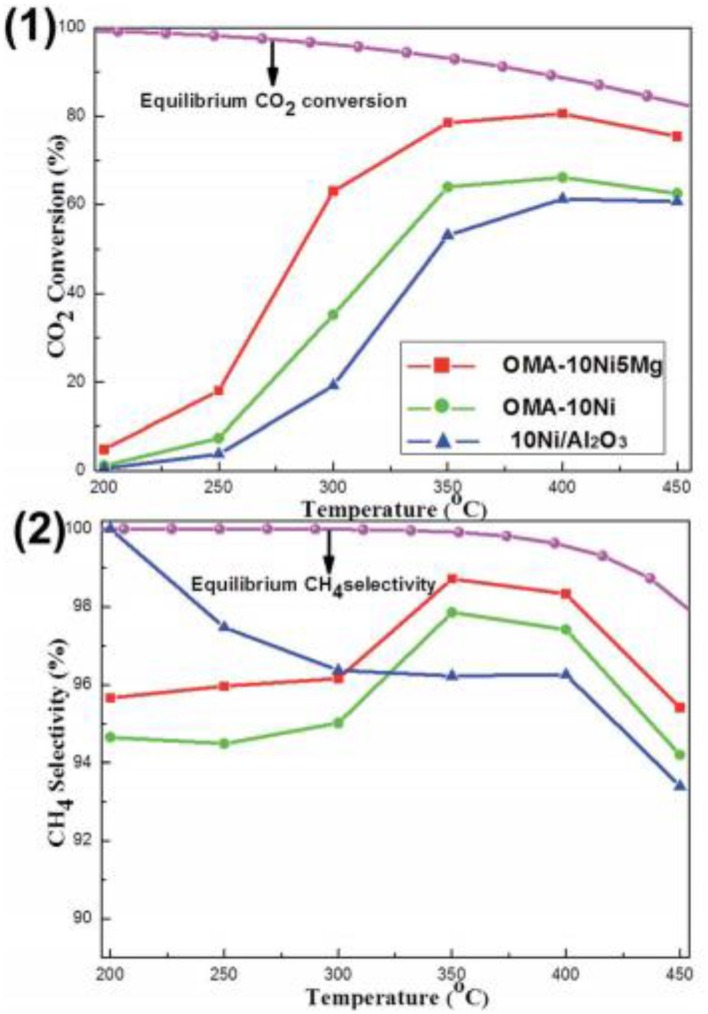
The curves of the (1) CO_2_ conversion and (2) CH_4_ selectivity vs. reaction temperature over OMA-10NixMg and 10Ni/Al_2_O_3_ catalysts; reaction condition: H_2_/CO_2_ = 4, GHSV = 15,000 mL g^−1^ h^−1^, 1 atm. Reproduced from Xu et al. (2017b) with permission from Royal Society of Chemistry.

As for the MgO, it has been extensively investigated as the basic modifier as the Ni based catalysts toward the CO_2_ methanation reaction (Ye et al., 2019). The catalytic activity of the catalyst can be enhanced by adding a low concentration of Mg because the chemisorption and dissociation of CO_2_ is promoted (Tan et al., 2019). Meanwhile, the addition of MgO can also improve the dispensability and improving the oxidizing environment around the Ni particles in the catalyst. Thereby, the serious agglomeration of metallic Ni active centers and carbon deposition over the catalyst surface can be effectively prevented (Al-Fatesh et al., 2017; Feng et al., 2018; Vidal Vázquez et al., 2018). In order to further investigate the role of Mg in depth, the researchers have extensively studied the catalytic activity of different catalysts doped with MgO. Li X. et al. (2019) found that MgNiO_2_ could be formed by co-impregnation in Ni-Mg/coconut shell carbon (CSC), which could enhance the metal-support interaction and improve the high temperature stability of Ni. Furthermore, Xu et al. (2017b) found that the addition of MgO could evidently increase the surface alkalinity of the catalyst by forming various types of basic centers with different intensities. As shown in Figure 10, the CO_2_ conversion of Ni-Mg/ordered mesoporous alumina matrix (OMA) was obviously higher than that of Ni/OMA. In addition, Guo and Lu (2014) reported that the introduction of MgO would greatly affect the structure of the catalyst and fully expose the Ni active center with coordination unsaturation to promote the transformation of reactants (H_2_ and CO_2_). However, excessive Mg doping can also cause blockage of the active center of the catalyst, resulting in the decrease of the catalytic activity (Li et al., 2014; Xu et al., 2017b).

**Figure 11:**
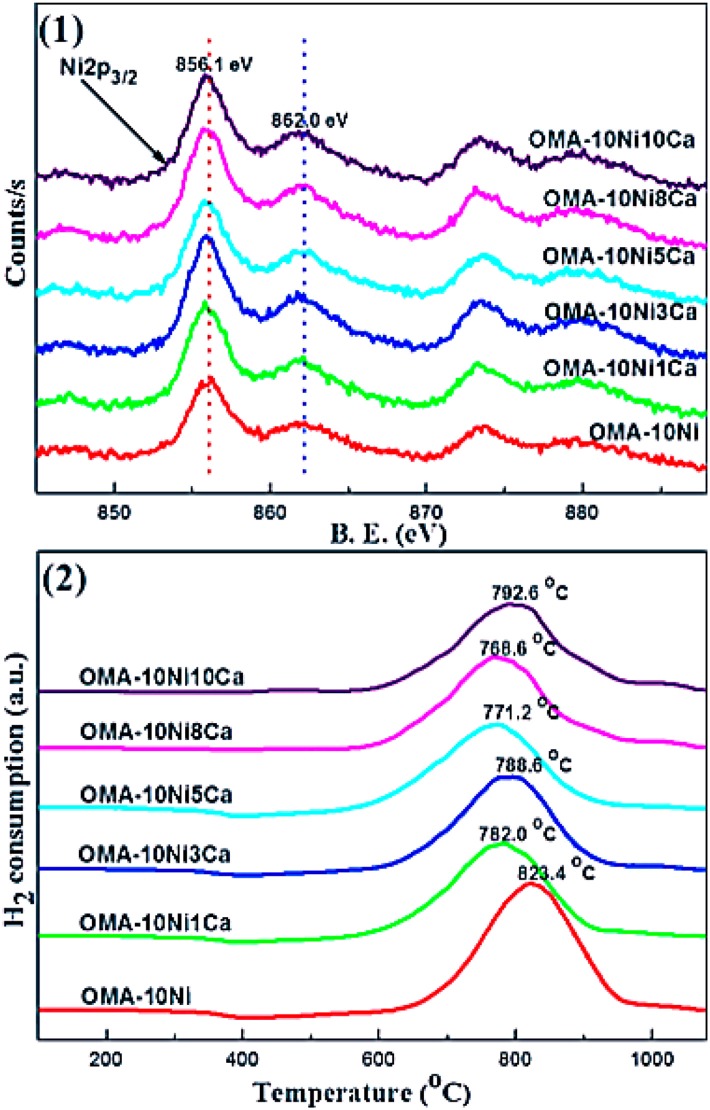
(1) Ni 2p XPS and (2) H_2_-TPR profiles of the as-prepared OMA-10NixCa catalysts. Reproduced from Xu et al. (2017a) with permission from Elsevier and Copyright Clearance Center.

CaO can intensify the chemisorption of the CO_2_ and then decrease the activation energy of CO_2_ as the basic modifier (Pan et al., 2014). Besides, it was reported that CaO increased the number and intensity of the basic sites over 15Ni/activated carbon (AC) catalyst (Feng et al., 2016). Therefore, the catalytic performance at low reaction temperature could be promoted. Furthermore, Xu et al. (2017a) found that Ca could increase the wall thickness of mesoporous channels of the catalyst, which could improve the thermal stability of these materials. Meanwhile, although Ca modifier had no effect on the chemical coordination environment of the Ni^2+^ cation, it could promote the reduction of Ni species by inhibiting the formation of NiAl_2_O_4_ spinel as shown in Figure 11.

##### Transition metals

Transition metals, such as Ti, V, Mn, Fe, Co, and Cu, have unique acid-base and redox properties and widely investigated as the modifier of Ni based catalysts toward CO_2_ methanation. For example, Yuan et al. (2018) further found that transition metal Re could significantly reduce the activation barrier of C-O bond cleavage, which was usually considered as the rate-determining step of methanation reaction. Therefore, Re dopant could accelerate the CO_2_ methanation process and thus improve low-temperature catalytic performance. Besides, it has been reported that the addition of the transition metal to Ni/Al_2_O_3_ can significantly change the electronic structure of the catalyst and affect the chemical properties of the Ni clusters of the catalyst (Shadravan et al., 2018; Long et al., 2019).

**Figure 12:**
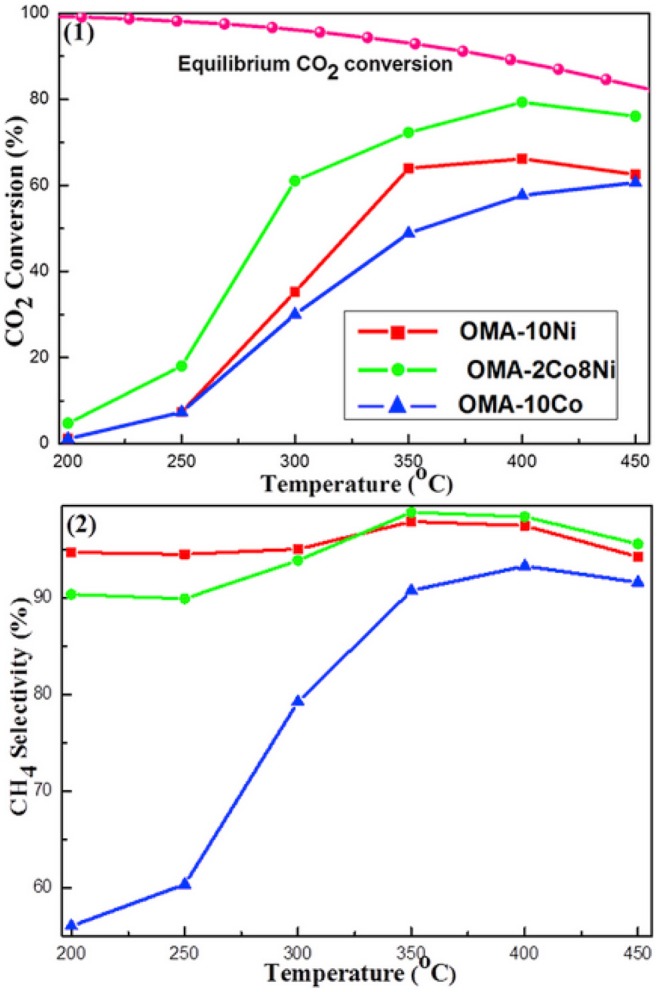
The curves of the (1) CO_2_ conversion and (2) CH_4_ selectivity vs. reaction temperature over OMA-10Ni, OMA-2Co8Ni, and (3) OMA-10Co catalysts; reaction condition: H_2_/CO_2_ = 4, GHSV = 15,000 mL g^−1^ h^−1^, 1 atm. Reproduced from Xu et al. (2018) with permission from Elsevier and Copyright Clearance Center.

In terms of Co, it has good CO_2_ activation ability at low temperature and can promote the uniform dispersion of active metal on catalyst (Xu L. et al., 2019). Xu et al. (2018) found that Co could improve the catalytic effect of Ni-based catalysts at low temperature. As shown in Figure 12, Ni-based catalyst doped with cobalt displayed much higher CO_2_ conversion than Ni, Co monometallic counterparts. This phenomenon may be ascribed to the synergistic effect between Co and Ni, which could make the catalyst resistant to thermal agglomeration (Siang et al., 2018). Liu et al. (2018b) found that Ni-Co/Al_2_O_3_ reduced the activation energy of the reaction and increased the catalytic activity compared with Ni/Al_2_O_3_. Furthermore, it was reported that the addition of Co could increase the reducibility of Ni based catalyst at low temperature and improve Ni dispersion over the support, which had a positive impact on the catalytic activity (Alrafei et al., 2019).

In recent years, Mn has been considered as an effective promoter in the CO_2_ methanation reaction. They have strong resistance to thermal sintering because the surface oxygen intermediates produced by manganese can react with the surface carbon deposition, inhibiting the formation of Ni carbide (Rahmani et al., 2014). Besides, the interaction between Mn and the oxide phase as well as the increased CO_2_ adsorption capacity can improve the catalytic activity of the catalyst (Le et al., 2019a). Zhao et al. (2016b) also found that the addition of Mn increased the alkaline site of the catalyst, which improved the CO_2_ chemisorption capacity and low-temperature catalytic activity of the catalyst. Besides, the oxygen vacancy of catalyst can promote the chemisorption and dissociation of CO_2_ during the process of methanation. Therefore, the doping of Mn can promote the activation of CO_2_ by generating more oxygen vacancies (Burger et al., 2018).

The low-temperature catalytic activity of Ni-Fe alloy catalyst is usually higher than those of the single-metal Ni reference counterparts, especially at higher pressure due to the optimal CO_2_ dissociation energy (Mutz et al., 2018). Simultaneously, with the addition of Fe, the stability of the catalyst, the conversion of CO_2_ and the selectivity of CH_4_ are all improved, indicating that Ni and Fe have synergistic effects (Mutz et al., 2017). In the view of the reaction mechanism, the addition of Fe could facilitate the formation of hydrocarbon, enhance CO_2_ adsorption capacity, and accelerate the reaction rate (Li et al., 2018d). It was reported that the promoting effect of the Ni/Fe ratio on the reducibility of the catalyst became obvious and the reducibility of the catalyst gradually became better with the increase of the Fe loading (Burger et al., 2018). Ren et al. (2015) also reported that both the appropriate electronic environment and the enhanced reductive properties of Fe were key factors in the enhancement of low-temperature catalytic performance toward CO_2_ methanation.

##### Rare earth metal oxides

The rare earth metal mainly includes lanthanide and actinide elements, such as Y, La, Ce, Pr, Nd, Sm, and so on. Compared with alkaline earth elements, rare earth elements with a unique electronic structure can regulate the electronic properties of active centers (Xu et al., 2017c; Fang et al., 2018). Fang et al. (2018) also found that the presence of rare earth metal could promote the reducibility of the Ni^2+^ species and greatly increase the number of surface basic sites. Furthermore, they are also commonly used as the lattice defect additives, which can make the arrangement of the Ni active center become irregular. As a result, the active crystallites exhibit more lattice defects, thereby finally making the catalyst expose more active centers to the gaseous reactants (He et al., 2015).

**Figure 13:**
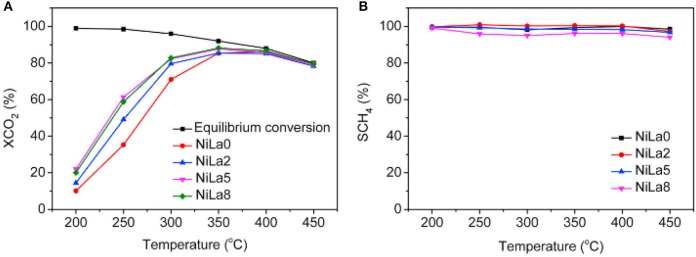
**(A)** CO_2_ conversion and **(B)** CH_4_ selectivity vs. temperature for the NiLax (x = 0, 2, 5, 8) catalysts. Reproduced from Zhang G. et al. (2018) with permission from Elsevier and Copyright Clearance Center.

**Figure 14:**
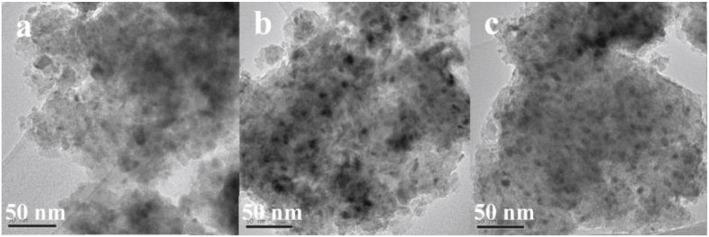
TEM images of the reduced catalysts: **(a)** NiLa0, **(b)** NiLa2, **(c)** NiLa5. Reproduced from Zhang G. et al. (2018) with permission from Elsevier and Copyright Clearance Center.

The doped La can endow the catalyst with moderate basicity and improve the CO_2_ adsorption capacity of the catalyst (Garbarino et al., 2019). As a result, the low-temperature catalytic activity and catalytic stability can be greatly improved owing to the enhancement in surface basicity and the dispersion of the Ni active centers (Wierzbicki et al., 2016; Liang et al., 2019c). In addition, La can have a positive effect on modifying the electronic environment around the metallic Ni active centers (Hu and Urakawa, 2018; Branco et al., 2020). Zhang et al. (2018a) prepared a La-doped Ni/Mg-Al used as the catalyst toward CO_2_ methanation. As shown in Figure 13, the obtained catalyst was provided with enhanced catalytic activity after doping an appropriate amount of La, especially under low temperature conditions. This phenomenon could be ascribed to the formation of surface free hydrogen promoted by La, which played the critical role in the process of removing nickel carbonyl on the surface. Besides, it could be observed in Figure 14 that the dispersion of Ni over the reduced catalyst became better with the increase of La loading amount. In addition, Quindimil et al. (2018) also found that an appropriate amount of La could obviously improve the reducibility and dispersion of Ni species. As a result, the number of accessible basic sites and active centers over the catalyst surface also increased owing to the enhancement of the dispersion of Ni.

As regards Ce, it has been considered as an important additive of Ni based catalyst toward CO_2_ methanation because it is beneficial to the improvement of various properties of the catalysts, such as the redox and dielectric properties, the stability of the catalyst, the activation of CO_2_ (Bian et al., 2015; Zhou G. et al., 2016; Movasati et al., 2017). For example, Liu H. et al. (2012) studied the effect of CeO_2_ on Ni-Al_2_O_3_ catalyst toward CO_2_ methanation process. They found that the addition of CeO_2_ could improve the thermal stability of alumina support. Specifically, the improvement of the catalytic performance of Ce-doped catalysts was mainly related to the significant improvement of Ni dispensability and the increase of the oxygen vacancy on the catalyst surface (Guilera et al., 2019). Besides, the addition of Ce could improve the catalytic activity of the catalyst by promoting the dispersion of the metallic Ni on the support. Bacariza et al. (2018b) reported that the CO_2_ conversion and CH_4_ selectivity over the Ni-Ce/Zeolite catalyst were significantly enhanced and it remained active at low temperature after the addition of Ce, which was mainly attributed to the effect of Ce on the reducibility of Ni species and the high permittivity of Ce.

In the case of Y dopant, it was reported that oxygen vacancies would be generated after its introduction (Qin et al., 2019). Besides, the strong interaction between the oxygen vacancies and the oxygen in the CO_2_ could weaken the strength of the C=O bond in CO_2_ molecule, which finally would inhibit the production of carbon deposits and promote the conversion of CO_2_ to methane at low temperature (Liang et al., 2019b). Therefore, Y displays an important role in improving catalytic activity. In addition, the Y incorporated catalysts usually exhibit excellent structural properties, such as large specific surface area, big pore volume, and narrow average pore size (Hwang et al., 2012). Takano et al. (2016) reported that the activity of the Y-doped Ni/ZrO_2_ catalyst was higher than that of the Ni/ZrO_2_ catalyst at all the doping concentration.

##### Noble metals

**Figure 15:**
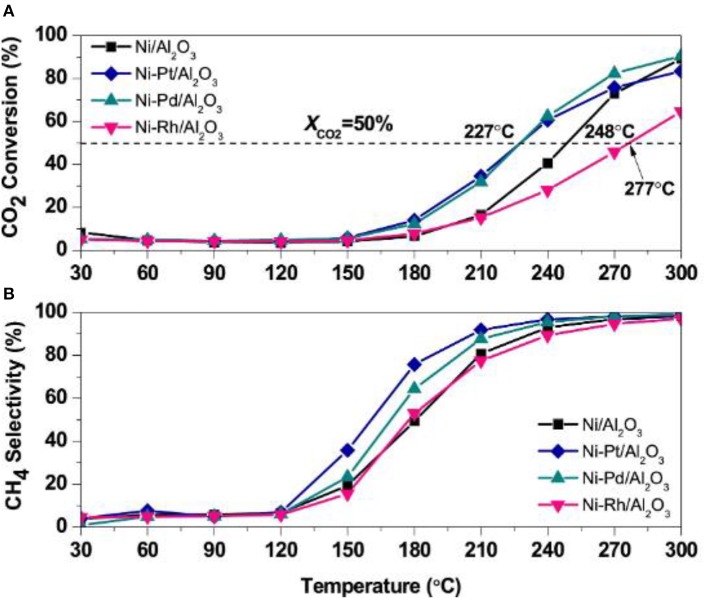
**(A)** CO_2_ conversion, and **(B)** CH_4_ selectivity profiles obtained during TPRea runs on Ni/γ-Al_2_O_3_, Ni-Pt/γ-Al_2_O_3_, Ni-Pd/γ-Al_2_O_3_, and Ni-Rh/γ-Al_2_O_3_ (CO_2_:H_2_ = 1:4, GHSV = 5,700 h^−1^). Reproduced from Mihet and Lazar (2018) with permission from Elsevier and Copyright Clearance Center.

Noble metals, such as Ru, Pd, and Pt, can evidently promote the dispersion and stabilization of Ni nanoparticles in the catalyst, which can contribute to the enhancement of the catalytic activity at low temperature. Besides, the precious metal can not only change the H_2_ adsorption capacity of the catalyst, but also provide more active centers for adsorbing and activating hydrogen (Kim et al., 2015). Mihet and Lazar (2018) carefully studied the catalytic effects of Rh, Pd, and Pt. Figure 15 demonstrated catalytic activity of catalysts. As shown, the CO_2_ conversion and CH_4_ selectivity over the Ni/Al_2_O_3_ catalysts doped with Pt or Pd were significantly higher than those without modification. Meanwhile, the doping of noble metal could also increase the interface between the metal and support, further enhancing the H_2_ chemisorption capacity. In addition, Zamani et al. (2015) reported that the addition of noble metals could further increase the reducibility of the catalyst and enhance the activation of CO_2_. Therefore, the doping of the precious metal is a significance method of enhance the catalytic performance (Ocampo et al., 2011).

In the case of Ru, the impregnation of its generally helps to reduce the particle size of the Ni active centers and thereby increase the surface area of the metallic Ni (Polanski et al., 2017). The high dispersion and narrow distribution of Ni-Ru nanoparticles over the support are beneficial to increasing the activity and stability of the catalysts toward CO_2_ methanation (Shang et al., 2018; Navarro-Jaén et al., 2019). Sharma et al. (2011) found that Ru could accelerate the decomposition rate of carbonate reaction intermediate and inhibit the formation of sintering of the catalyst. Besides, the synergistic effect of the bimetallic Ni-Ru can improve the chemisorption capacity of the catalyst for H_2_, but Ru has no significantly promoting effect on the adsorption capacity of CO_2_ (Liu et al., 2018a).

For the single metal catalysts, Pd based catalyst often displays much higher catalytic activity but lower CH_4_ selectivity than the Ni-based catalyst (Beaumont et al., 2014). Therefore, it was believed that the bimetallic Ni-Pd catalyst could achieve better catalytic performance due to the synergistic effect between Pd and Ni (Oemar et al., 2015). Li B. et al. (2019) found that the catalytic performance of the bimetallic Ni-Pd/SBA-15 catalyst was better than that of the corresponding monometallic Ni and Pd counterparts. The strong interaction between Ni and Pd existed on the catalyst by forming bimetallic Ni-Pd alloy and the charge transfer from Pd to Ni atoms caused surface Ni atoms to receive more negative charge. Specifically, the charge transference from Pd to Ni atoms caused the surface Ni atoms to accumulate lots of negative electrons, which can promote the adsorption and dissociation of CO_2_ (Steinhauer et al., 2009).

#### Composite Additives

Recently, the composite additives have been gradually attracted more and more attention due to their outstanding catalytic performances because the composite additives can combine the advantages of different additives together to achieve enhanced catalytic performance toward CO_2_ methanation (Toemen et al., 2016). Generally, the catalysts doped with composite additives usually exhibit higher thermal sintering resistance and better stability than the counterpart with single additive due to the synergistic effect between different additives (Frontera et al., 2018).

**Figure 16:**
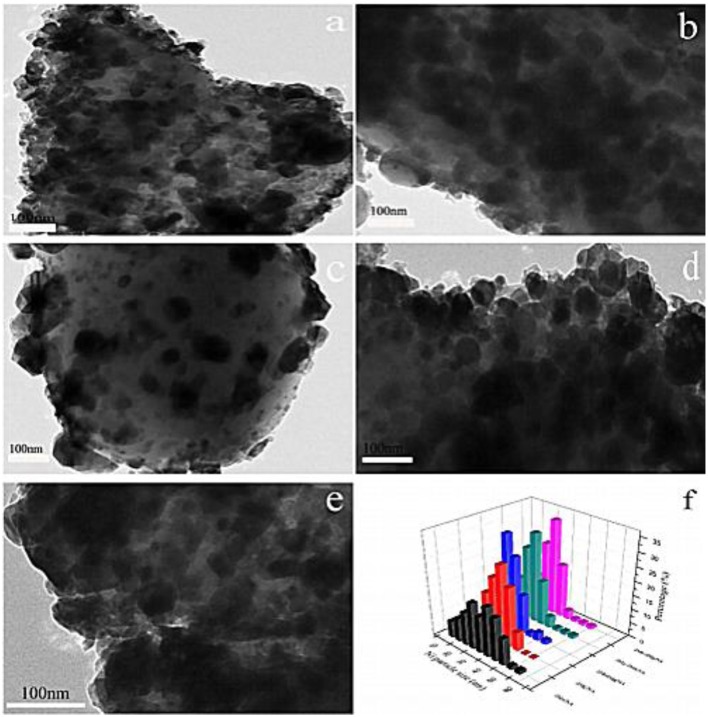
HRTEM images of **(a)** 2 Mn, **(b)** 2 Mg, **(c)** 2Mn2Mg, **(d)** 2Mg-2Mn, **(e)** 2Mn-2Mg, and **(f)** particle distribution of reduced catalysts. Reproduced from Zhou et al. (2018) with permission from Elsevier and Copyright Clearance Center.

It was reported that the combination of the electronic additive Mn and the lattice-defective additive Mg could greatly promote the dispersion of the active component, hinder the thermal sintering of the active component, and enhance the strong interaction between the support and metallic Ni, thereby promoting the CO_2_ methanation reaction (Rahmani et al., 2014; Tan et al., 2019). Meanwhile, the electron transference between the composite additives has a positive effect on the activation and dissociation of CO_2_ on the catalyst surface (Ramezani et al., 2018). Besides, Zhou et al. (2018) found that the Ni particle size of the catalyst doped with Mn and Mg was significantly reduced compared to the catalysts with single additives. As shown in Figure 16, large Ni particles were presented with severely agglomerated on the Mn doped Ni/Al_2_O_3_ and Mg doped Ni/α-Al_2_O_3_. However, the surface of dual promoter doped catalysts were homogeneously covered with Ni particles, which would facilitate the activation of H_2_ and CO_2_ conversion. Thus, adding Mg and Mn additives was considered as an effective method to improve catalytic performance of Ni-based catalysts.

Ru and Mn composite additives can decrease the coke formation and then improve the stability of the catalyst during the CO_2_ methanation reaction (Kim et al., 2015). Besides, Ru and Mn promotes the adsorption and dissociation of CO_2_ because they can increase the surface active centers of the catalyst (Zamani et al., 2019). Wan Abu Bakar et al. (2015) compared the effects of Mn single additive and Ru-Mn composite additive on the catalyst, respectively. They found that Ru/Mn/Ni/Al_2_O_3_ exhibited better catalytic activity than Mn/Ni/Al_2_O_3_. The CO_2_ conversion of the Ru/Mn/Ni/Al_2_O_3_ catalyst could be as high as 99.7% at 300°C. Specifically, the high CO_2_ conversion was probably due to the Mn species which caused the removal of Cl ions from RuCl_3_ precursor and increased the density of active Ru oxide species on the catalyst which resulted in the high catalytic activity.

Mn-Fe has been described as efficient composite additives because both Mn and Fe could promote the adsorption and activation of CO_2_, which is considered as an important part of CO_2_ methanation (Rahmani et al., 2014; Li et al., 2018d). In addition, the modification of Mn can increase the density of the basic sites on the catalyst, further enhancing the low-temperature catalytic activity (Burger et al., 2018). The thermal stability of the catalyst can be intensified by doping Fe because the Ni-Fe alloy forming during the catalytic process allows Ni to be uniformly dispersed on the catalyst surface (Zagaynov et al., 2019). Furthermore, it was reported that the reduced Fe and Mn would interact with the metallic Ni phase, thereby affecting the equilibrium state between the Ni atoms and causing an alleviated reducibility of the active Ni phase.

### Summary

In general, the alteration of the supports and the addition of the additives have been the main research line of Ni-based catalysts. The researches on supports were mainly carried out from multiple perspectives such as increasing the surface area, changing the channel structure, and adjusting the pore diameter, which could benefit the dispersion of the metallic Ni active sites. The research of catalytic additives mainly included the above four parts, including the alkaline earth metal oxides, transition metals, rare earth metal oxides, and noble metals, which contributed to the activation of the CO_2_ at low temperature. The development of Ni-based catalysts with low-temperature catalytic activity should focus on the interaction of additives and supports in catalytic reactions in the future.

## Preparation Conditions of NI-based Catalyst

Generally, the physicochemical properties of the Ni based catalyst and its catalytic activity are greatly affected by the preparation conditions, such as the preparation method and calcination conditions (Bacariza et al., 2015). Specifically, calcination is considered as a decisive step in changing the performance of the catalyst. An increase in the calcination temperature can change the position as well as type of metallic Ni active centers on the support and decrease the porosity of the catalyst (Zhang et al., 2011). Besides, the preparation method also has significant effects on the properties of the catalysts, such as surface alkalinity, reducibility of the Ni species. It was reported that the reducibility of Ni species could be improved over the catalyst prepared by hydrothermal method (Guo et al., 2018). In addition, the Ni loading over the catalyst also greatly influences the catalytic performances. Specifically, only the moderate loading amount of Ni can significantly increase the catalytic activity and the thermal sintering of Ni particles will cause blockage of the pores of the support (Lechkar et al., 2018).

### Preparation Method

The dispersion of Ni active components and the metal-support interaction are closely related to the catalyst activity, which was strongly influenced by the preparation method. Therefore, preparation method of catalyst is an importantly influential factor to improve catalytic performance (Guilera et al., 2019). The specific advantages and disadvantages of the preparation methods are summarized in Table 2. Each method has its own unique advantage. The impregnation method has been considered as a cheap and efficient method, which mainly employs the capillary pressure to push the active components into the pore channels of the support (Romero-Sáez et al., 2018). Besides, the catalyst prepared by coprecipitation usually has a large specific surface area (Beierlein et al., 2019). The sol-gel method can promote the high dispersion of active components on the support (Moghaddam et al., 2018). Solution combustion is a simple and easy technique for the operation, where the oxidant is the metal nitrate and the reducing agent is the fuel. The fuel not only provides energy for the reaction, but also mixes with metal cations. Thus, the catalyst prepared by solution combustion method usually has good catalytic activity (Zhao et al., 2012; Jiang et al., 2018; Xanthopoulou et al., 2018).

**Table 2:** Specific advantages and disadvantages of the preparation methods.

**Preparation method**	**Advantages**	**Disadvantages**
Impregnation method	It is easy to operate.	The metal particles of the catalyst are unevenly distributed in the pores of the porous material.
Precipitation method	(1) It is easy to operate. (2) The size and distribution of the metal particles are relatively uniform.	PH value and temperature have greatly impacts on the catalyst.
Sol-gel method	(1) Outstanding stability. (2) Good catalytic performance. (3) High Ni dispersion.	Long response time.
Plasma method	(1) Reduce the particle size of the catalyst. (2) Improve the dispersion of Ni. (3) Enhance the interaction between the Ni and the support.	(1) Immature technology. (2) High requirements for operation parameters.
Urea hydrolysis method	Not affected by pH.	—
Ammonia evaporation method	Enhanced metal-support effect of the catalyst.	—
Microwave assisted method	(1) Short reaction time. (2) Uniform heating for the substances with same microwave absorbing properties.	—

**Figure 17:**
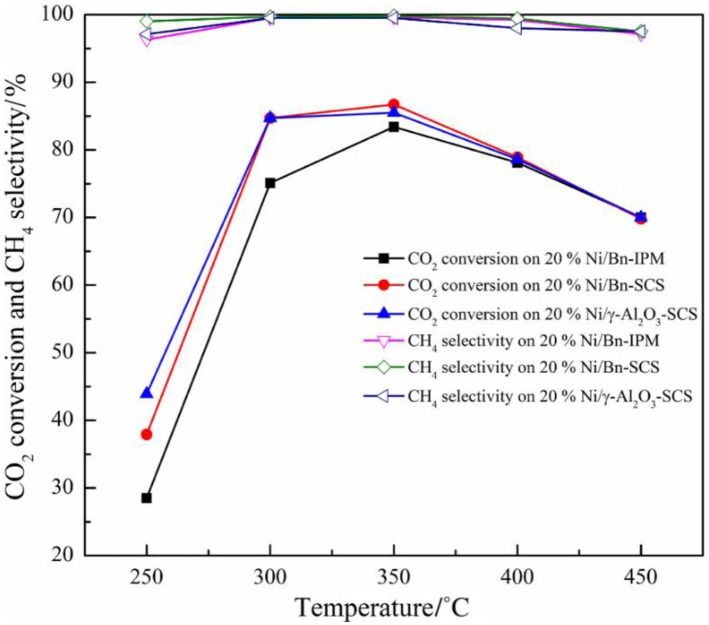
The catalytic activity of Ni/Bn-IPM, Ni/Bn-SCS, and Ni/γ-Al_2_O_3_ catalyst was for CO_2_ methanation. Reaction conditions: V(H_2_)/V(CO_2_) = 4:1, atmospheric pressure, GHSV = 3,600 ml·(g cat)^−1^ ·h^−1^. Reproduced from Jiang et al. (2018) with permission from Elsevier and Copyright Clearance Center.

Beierlein et al. (2019) prepared Ni-based catalysts by dry impregnation (DI), wet impregnation (WI), sedimentation precipitation (DP), and coprecipitation (CP) methods. For catalysts prepared with CP and DI, it was found that the pore size distribution was related to the mass fraction of Ni. As the comparison, the pore size distribution of the catalyst prepared by DP and WI was not affected by the Ni loading. The saturation concentration of Ni loading existed during the wet impregnation due to the limitation of solubility. The saturation concentration hindered the maximum Ni loading amount over the support. Therefore, the structure of the catalyst prepared by WI was not affected as the Ni loading increased. During the drying process of the impregnated catalyst, Ni species gradually entered the mesopores of the supports with the increase of the Ni loading. In the DP catalyst, nickel hydroxide did not form a precipitate in the solution. The increase in the Ni loading could greatly result in a significant increase in the Ni surface area, which in turn affected the catalytic activity in the form of CO_2_ conversion. In addition, if it is aimed to improve the dispersion of Ni species on the surface of the catalyst, the deposition precipitation method and the coprecipitation method will be considered as better candidates because the surface area of the catalyst the surface area of Ni nanoparticles prepared by the precipitation method are usually large, which is an important factor for promoting the CO_2_ conversion. Besides, Jiang et al. (2018) prepared Ni/bentonite catalyst by solution combustion method (SCS). Compared with the catalyst prepared by the impregnation method (IMP), Ni species were provided with higher dispersion, narrower particle size distribution, and larger specific surface area, thereby promising outstanding catalytic activity. As shown in Figure 17, the activity of the catalyst prepared by the SCS method was better than that of IMP catalyst.

**Figure 18:**
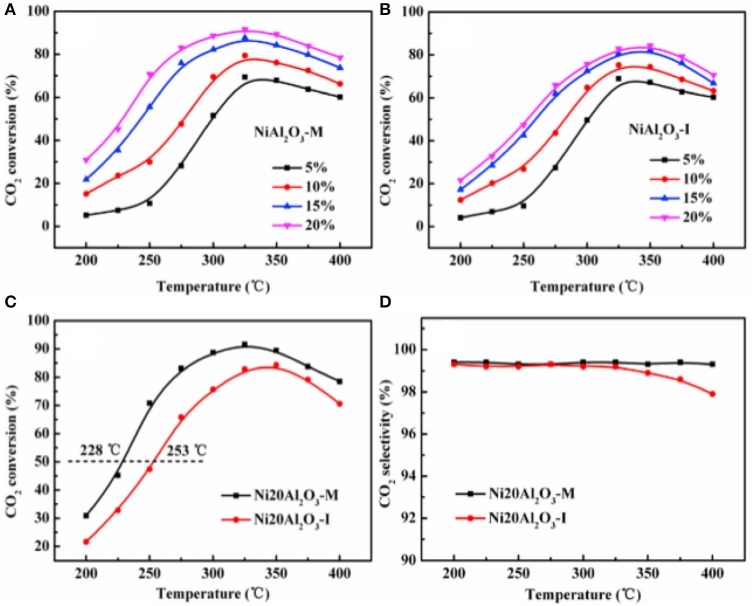
The CO_2_ conversion over the catalysts **(A–C)**: **(A)** NiAl_2_O_3_-M with different Ni loadings; **(B)** NiAl_2_O_3_-I with different Ni loadings; **(C)** Ni20Al_2_O_3_-M and Ni20Al_2_O_3_-I; **(D)** the CH_4_ selectivity over Ni20Al_2_O_3_-M and Ni20Al_2_O_3_-I. Reproduced from Song et al. (2017) with permission from Elsevier and Copyright Clearance Center.

**Figure 19:**
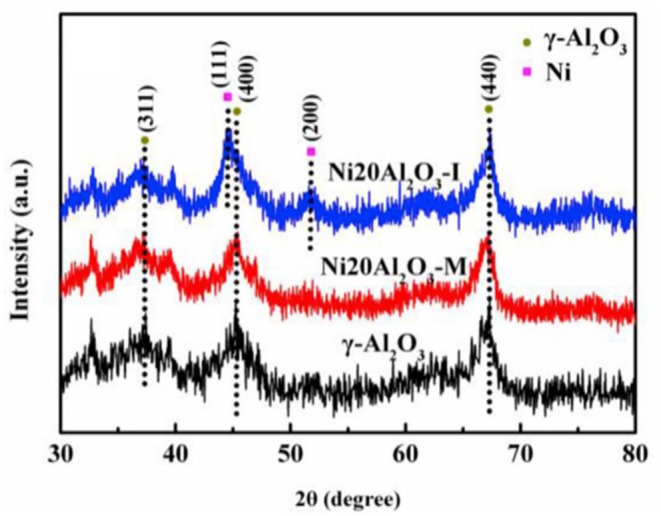
XRD patterns of Al_2_O_3_, Ni20Al_2_O_3_-M and Ni20Al_2_O_3_-I. Reproduced from Song et al. (2017) with permission from Elsevier and Copyright Clearance Center.

In recent years, the researchers have also investigated and developed new preparing methods by employing new technologies, such as microwave assisted method, plasma method, and urea hydrolysis method. Compared with the coprecipitation method, the catalyst prepared by the ultrasonic assisted coprecipitation method has a larger surface area and pore volume, and more uniform Ni nanoparticle distribution (Daroughegi et al., 2017). Daroughegi et al. (2019) found that the use of ultrasonic irradiation during precipitation could inhibit the agglomeration of Ni particles. Besides, the catalyst prepared by the oxalate method and the ammonia evaporation method also has excellent catalytic performance at a relatively low reaction temperature owing to the outstanding stability of the catalyst (Lo Faro et al., 2015; Ashok et al., 2017). The citric acid complication is superior to traditional impregnation because it can effectively reduce the particle size and promote the dispersion of Ni species, which is beneficial to the intrinsic activity of the catalyst (Tan et al., 2019). The plasma is composed of ionized ions, which is featured as high efficiency, less pollution, and simple operation, and high reactivity. A synergistic effect between the plasma and the catalyst allows the CO_2_ methanation reaction to proceed at a lower temperature (Biset-Peiró et al., 2019; Wierzbicki et al., 2019). Specifically, the textural properties, such as surface area and pore volume, of the Ni-based catalyst prepared by the plasma method will be better than those of traditional catalyst. Furthermore, it was reported that Ni based catalysts prepared by plasma have higher Ni dispersion and CO_2_ adsorption capacity (Xiong et al., 2019). Therefore, great efforts have been devoted to the study of plasma methods (Bian et al., 2015). Debek et al. (2019) used the low-pressure glow discharge plasma method to prepare a Ni-based catalyst, which could impede carbon deposition on the catalyst and enhance the stability of the catalyst under low pressure conditions. As regards the microwave assisted method, its main characteristic is that microwave is used as the heat source. As an energy-efficient technology, the microwave assisted method can shorten the reaction time because the microwave can simultaneously heat the bulk and surface parts of the catalyst from the molecular level (Jing et al., 2018). The microwave assisted method can also be combined with the coprecipitation method or sol-gel method, which can achieve better catalytic effects and performances (Dong et al., 2018). Song et al. (2017) investigated Ni based catalysts supported on Al_2_O_3_ prepared by impregnation (I) and microwave assisted methods (M). The CO_2_ conversion rates of NiAl_2_O_3_-M were higher than that of NiAl_2_O_3_-I at the equal amount of Ni loadings in Figure 18, which may be attributed to the high dispersion of Ni nanoparticles. As shown in Figure 19, the metallic Ni diffraction peaks could not be observed over Ni2OAl_2_O_3_-M catalyst, indicating that the Ni particles were highly dispersed in nano size state, lower than the XRD detection limit (3.0 nm). As a comparison, the Ni20Al_2_O_3_-I catalyst presented much higher metallic Ni diffraction peaks, suggesting the worse dispersion of metallic Ni particles. Therefore, the microwave method could facilitate the uniform distribution of Ni on the support. In addition, microwave assisted method contributes to greater catalytic stability and sintering resistance of the catalyst (Dong et al., 2018). As for the urea hydrolysis method, it uses urea as a precipitant and can react with metal precursors controllably at relatively low temperatures. As a result, the CO_2_ adsorption capacity of the catalyst prepared by the urea hydrolysis method is stronger than that of the catalyst prepared by the coprecipitation method (Zhang et al., 2018a). Layered double hydroxides (LDHs) materials are mixed hydroxides of di- and tri-valent metals, present in the brucite like layers with anions present between the layers (Wierzbicki et al., 2019). Ni based catalysts prepared by synthesizing LDH as precursor have attracted considerable interests for CO_2_ methanation because the catalytic activity of the catalyst was improved compared to Ni-based catalysts prepared by conventional methods like impregnation (Li et al., 2016). Specifically, the high sorption capacity for CO_2_ in the layered space was crucial for the methanation of CO_2_ reaction (Guo et al., 2016). In addition, it was reported that DLHs formed nano-oxides after appropriate thermal treatment whose strong interaction hindered Ni from sintering (Liu J. et al., 2015).

### Nickel Loading

**Figure 20:**
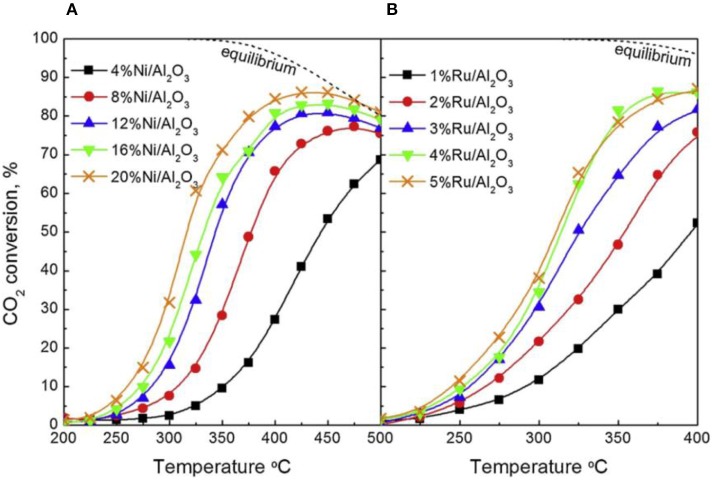
CO_2_ conversion as a function of reaction temperature for **(A)** Ni/Al_2_O_3_ and **(B)** Ru/Al_2_O_3_ catalysts. Reproduced from Quindimil et al. (2019) with permission from Elsevier and Copyright Clearance Center.

Recently, the influence of the metallic Ni loading content on the catalytic performance of different catalysts has been widely reported (Su et al., 2016). Within a certain range, the activity of the catalyst can increase with the increase of the Ni loading amount due to providing sufficient active centers. Besides, the metal loading amount also can affect the reduction property of the catalyst (Wierzbicki et al., 2017). Zhang Z. et al. (2019) found that the reverse water gas shift (RWGS) side reaction had a competitive relationship with the CO_2_ methanation reaction in the case of low nickel loading amount and the high nickel loading (around 25%) was beneficial to the enhancement of the CO_2_ methanation reaction. As shown in Figure 20, the CO_2_ methanation reaction gradually dominated with the increase of the Ni loading amount. They found that the catalyst could achieve the best activity for methanation and low yield of CO when the Ni loading was 20 wt%. Therefore, it could be concluded that the increase of active sites (Ni^0^) was beneficial to the achievement of high CO_2_ conversion. However, when the nickel loading amount exceeded a certain range, further increasing the loading amount would cause seriously thermal agglomeration of the metallic nickel active centers, thereby destroying the structure of the catalyst (Quindimil et al., 2019). Ocampo et al. (2011) found that the charge of nickel cation and its coordination state in the crystal lattice affected its solubility in the CeO_2_-ZrO_2_ structure. In the CZ bimetal architecture, it would compete with Zr^4+^, resulting in a decrease in NiO concentration on the catalyst surface when the concentration of Ni^2+^ increased. Furthermore, they found that high Ni species loading would result in the formation of abundant Ni active centers and improved low temperature CO_2_ methanation performance (Lin et al., 2019). Therefore, the reactivity of the catalyst varied with the loading of the active component.

It was reported that the loading amount of Ni supported on the catalyst has a significant effects on both the dispersion of Ni on the catalyst surface and metal-support interaction, which in turn affects the catalytic behavior. Overall, the dispersion of the metallic Ni active centers decreases as the loading increases (Quindimil et al., 2019). Ali Lechkar et al. (2018) preliminarily found that at least two different CO_2_ methanation reaction sites existed in a Ni-based catalyst, which was identified as Ni crystals from NiO reduction and the Ni atoms surrounded by oxygen atoms in the alumina crystal lattice. More reactive sites can be associated with readily reducible α and β NiO species, while less reducible ɤ-NiO species may be associated with less reactive sites. They also found that lots of ɤ-type NiO species with less reducibility existed on the catalyst when the Ni loading was low. However, β-type materials with more reducibility and reactivity are dominant under conditions of high nickel loading. β-type NiO species play a pivotal role as the main active centers for the methanation reaction (Alihosseinzadeh et al., 2015). Therefore, the effect of metallic Ni loading amount on the catalytic performance of the catalyst can be attributed to the increase in the number of active centers over the catalyst surface.

### Calcination Temperature

It was well-known that the activity of Ni-based catalysts could largely depend on the calcination temperature because it could change the dispersion of active metal particles and influence the activation of the catalytic center of the catalyst, thereby affecting the activity of the catalyst (Al-Fatesh and Fakeeha, 2012; Özdemir et al., 2014; Al-Fatesh et al., 2019).

**Figure 21:**
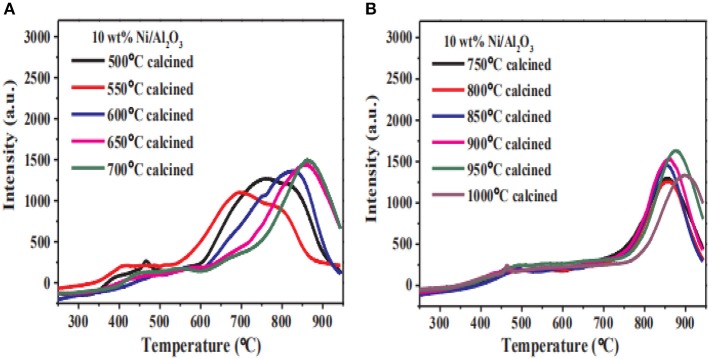
H_2_-TPR and reduction degree for the Ni/Al_2_O_3_ catalysts with different calcination temperature (T). **(A)** T = 500, 550, 600, 650, 700°C and **(B)** T = 700, 750, 800, 850, 900, 950, 1000°C. Reproduced from Zhang C. et al. (2018) with permission from Elsevier and Copyright Clearance Center.

The local structure of the catalyst framework would be destroyed during calcination process, especially at high temperature. Therefore, the pore size of the catalyst decreases with the calcination temperature increasing due to the thermal shrinkage of the framework (Zamani et al., 2019). Commonly, the lower calcination temperature is beneficial to preventing the agglomeration of Ni species. Sun et al. (2007) found that increasing the calcination temperature would cause a sharp decrease in the surface area of the structure, resulting in the collapse of the porous structure. In addition, the Ni particles could migrate from the outer surface to internal positions with the increase of the calcination temperature, which is usually considered as the main reason for the improvement in the catalytic activity. However, the increase in the calcination temperature can also reduce the dispersion of the Ni species, resulting in a lower catalytic activity (Bacariza et al., 2015). Zhang C. et al. (2018) studied the reducibility of Ni/Al_2_O_3_ catalysts at different calcination temperatures. As shown in Figure 21 with the increase of the calcination temperature, the main reduction peak of the catalyst shifted to high temperature. When the calcination temperature was higher than 650°C, the reduction degree of the catalyst gradually decreased. This indicated that the increase of calcination temperature intensified the strong metal-support interaction between the nickel and the alumina support, which could make the reduction of the Ni species in H_2_ stream very difficult.

### Summary

Overall, the preparation strategies and conditions can directly affect the catalytic performance of the catalyst by influencing the morphology and promoting the dispersion of metallic Ni active components. Besides, the calcination temperature and the metallic Ni loading amount are commonly used to improve catalyst performance. Therefore, great efforts will still be devoted to promote the low-temperature activity of the catalysts by employing different preparation methods.

## The Influences of Reaction Conditions for NI-based Catalyst Toward CO_2_ Methanation

The reaction conditions of the Ni-based catalyst, such as reaction temperature, gas hourly space velocity, etc., have important influences on the CO_2_ methanation process. Younas et al. (2018) used a quadratic model to correlate variable parameters such as methanation temperature, humidity, and catalyst mass with the effect of CO_2_ concentration. Their studies showed that the reaction temperature and humidity had an important impact on CO_2_ methanation.

**Figure 22:**
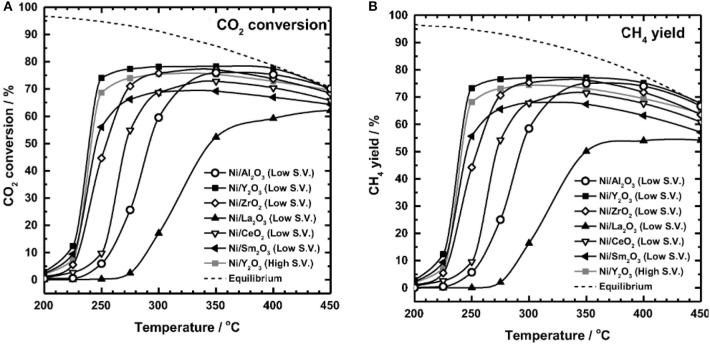
**(A)** CO_2_ conversion, and **(B)** CH_4_ yields in CO_2_ methanation over 10 wt.% Ni/metal oxide catalysts (Al_2_O_3_, Y_2_O_3_, ZrO_2_, La_2_O_3_, CeO_2_, Sm_2_O_3_). Reaction gas: 10%CO_2_-40%H_2_-50% N_2_. Low S.V.: 20,000 l kg^−1^ h^−1^. High S.V.: 30,000 l kg^−1^ h^−1^. Reproduced from Muroyama et al. (2016) with permission from Elsevier and Copyright Clearance Center.

The reaction temperature is considered as the dominant factor in the activity of Ni-based catalysts (Stangeland et al., 2017b). Ni-based catalysts usually have low CO_2_ conversion at low temperatures because the dissociation of CO_2_ chemical bonds requires higher activation energy. With the increase of the temperature, the CO_2_ molecule can acquire the desired activation energy and achieve the improvement in reactivity. However, the metallic Ni active centers easily suffer thermal sintering at high reaction temperature (Ma et al., 2011; Stangeland et al., 2017a; Tada et al., 2017). Muroyama et al. (2016) studied the effect of temperature on the catalytic performances over different catalysts. As shown in Figure 22, most of the investigated catalysts performed their respective maximum CO_2_ conversion and CH_4_ selectivity at 300–350°C. However, further increasing the reaction temperature would cause the decrease of the catalytic activity and CH_4_ selectivity. From the viewpoint of thermodynamics, the high temperature and exothermic feature of CO_2_ methanation process can affect the thermodynamic equilibrium and deactivation of the catalyst. The high temperature facilitates the reverse water gas shift reaction and hinders the CO_2_ methanation reaction (Mutz et al., 2015; Pastor-Pérez et al., 2019).

In addition to the reaction temperature, the pressure and humidity are also considered as important ways to optimize the CO_2_ methanation process. Mutz et al. (2017) found that the CO_2_ conversion experienced significant increase in the temperature range of 250–450°C when the pressure increased from 1 bar to 10 bar. The phenomenon could be ascribed to deposited carbon amounts affected by the pressure. Carbon deposition is a significant factor affecting catalyst performance. Thus, the catalytic reactor should be operated at elevated pressure to avoid carbon formation (Jurgensen et al., 2015). Besides, it was found that increasing the humidity could increase the CH_4_ selectivity of the catalyst because the presence of water vapor had the positive effect of inhibiting the reverse water gas shift (RWGS) reaction and thereby promoting the conversion of CO_2_ to CH_4_ (Jiménez et al., 2010). However, the CO_2_ conversion rate and the CH_4_ selectivity are lowered with excessive humidity because the affluent water molecules can cover the metallic Ni active center of the catalyst (Zhang et al., 2018b). For example, Aziz et al. (2015) reported that the presence of water vapor had a negative effect on the catalytic of 5Ni/MSN and alsocaused the structure collapse of MSN. Specifically, the structure collapse may be ascribed to the mechanochemical reaction through the hydrolysis of Si-O-Si bond in the presence of adsorbed water due to its hydrophilicity (Batista et al., 2005). The negative effect was possibly due to the formation of CO_2_ through the water gas shift (WGS) reaction between intermediate CO and the excess of water. In addition, water vapor may cause dilution of the gaseous reactants, inhibiting the interaction of H_2_ with CO_2_ (Younas et al., 2018).

Various methanation reactors have also been developed to avoid the thermal sintering of the Ni-based catalysts and improve the catalytic performance. Common reactors include fixed-bed reactors and fluidized-bed reactors (Zimmermann et al., 2019). Fixed-bed reactors are widely used in industrial applications. The main concern of fixed-bed reactor research for CO_2_ methanation is the control of reactor temperature (Rönsch et al., 2016). Structured reactors are under development to overcome this drawback, which has better heat transfer capacities and lower pressure drops than traditional fixed bed reactors (Kreitz et al., 2019). Compared with fixed-bed reactors, fluidized-bed reactors have a higher CO_2_ conversion rate, which are closer to the point of thermodynamic equilibrium. Nevertheless, the fluidized-bed reactor also possesses its own disadvantages. Specifically, the fluidization of catalyst particles requires high mechanical stress of the particles and reactor walls, which will cause the loss of the catalyst and shorter reactor life Liu J. et al. (2015).

Generally, the development trend of catalysts should focus on the improvement of the low-temperature catalytic activity and the maintenance of the high catalytic stability in the future based on the previously investigated reaction.

## Catalytic Application of NI-based Catalysts in Industrialization of CO_2_ Methanation

With the continuous improvement of the awareness of environmental protection and the increasingly urgent task of reducing greenhouse gas emissions, the research on industrialization of CO_2_ methanation has been vigorously launched in the 21^st^ century. The catalysts of the industrial CO_2_ methanation is summarized in Table 3 (Golosman and Efremov, 2012). The Ni-based catalysts are generally selected for industrial methanation technology (Kopyscinski et al., 2009). However, various challenges still exists in industrial applications of Ni-based catalysts. Specifically, CO_2_ mathanation is a strongly exothermic reaction, which would cause thermal sintering of the metallic Ni-based catalyst (Kopyscinski et al., 2009). In addition, the stress by long-term operation and impurities in the feed could deactivate the catalyst (Wolf et al., 2019). Therefore, many scholars have made great efforts to address these issues. Burger et al. (2018) synthesized Fe- and Mn- promoted co-precipitated Ni-Al catalysts. The focus on this work was placed on NiAl-based catalysts due to their advantages in metal costs and excellent catalytic performance. Besides, it was reported that the core-shell structure could make Ni nanoparticles dispersed in the porous silicon shell and the surface would not be easy to generate carbon deposition, thus promising a long lifespan of several months (Le et al., 2019b). In addition, Wolf et al. conducted a systematic study of co-precipitated Ni-Al catalysts in order to investigate sulfur poisoning (Wolf et al., 2019). It was found that sulfur poisoning was ascribed to site blockage rather than electronic effects. Overall, the development of Ni-based catalysts in the application of industrial CO_2_ methanation is still immatureand the related researches are not enough, which demand further investigation, such as sulfur poisoning.

**Table 3:** Summary of the Ni-based Catalysts for industrial CO_2_ methanation.

**Catalyst brand**	**Main active component**	**Operating temperature/**°**C**	**Pressure/Mpa**	**Lifetime**	**Conversion**
CRG-LH	Ni	250–700	1-6	2–3 years	≥98%
RANG-19	Ni	200–450	up to 4	—	—
RANG-19PR	Ni	200–450	up to 4	—	—
MCR-2X	Ni	250–700	up to 8	2–3 years	≥98%
CI-85	Ni	260	1-6	3–4 years	≥98%

## The Mechanism of CO_2_ Methanation Over NI Based Catalysts

CO_2_ methanation has great potential to break through the bottleneck of CO_2_ immobilization and resource utilization. Therefore, this reaction has recently received more and more attention due to its important strategic significance. The rational design of the Ni-based catalyst used in this reaction mainly depends on the determination of the metallic active centers and surface reaction intermediates (Zhou et al., 2017; Liang et al., 2019a).

Generally, the performance of CO_2_ in methanation reaction can be divided into two steps. Specifically, the first step is that CO_2_ reacts with the catalyst to form the carbonaceous intermediates and the following step is that the carbonaceous intermediates on the catalyst surface reacts with hydrogen species to form methane. However, the intermediates produced by the CO_2_ methanation reaction is currently no uniform consensus of the interpretation (Yang Lim et al., 2016). Some researchers believe that CO is the most likely intermediate for methanation (Westermann et al., 2015). CO_2_ absorbs on the surface of the catalyst dissociates to form CO, then CO dissociates into C and O species and then C hydrogenates to generate CH_4_ (Miguel et al., 2018; Li Y. et al., 2019). In the first stage of the reaction, the stability of the adsorption of CO on the surface of the catalyst is the key step to determining whether CO is desorbed or further reduced. The equilibrium between the formation of adsorbed carbon and its removal from surface hydrogen reactions affects the conversion of CO_2_ (Jalama, 2017). Therefore, co-dissociation is a decisive factor affecting the rate of residual reduction steps (Li Y. et al., 2019).

**Figure 23:**
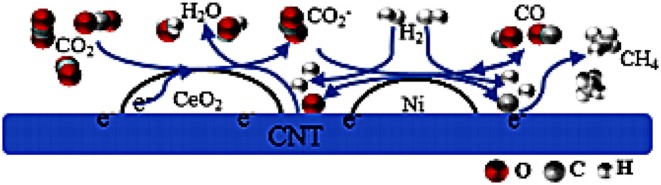
Reaction mechanism on the cerium promoted nickel catalyst supported on CNTs for CO_2_ methanation. Reproduced from Wang W. et al. (2016) with permission from Elsevier and Copyright Clearance Center.

Another viewpoint that CO is not the CO_2_ methanation reaction intermediate also has been put forward. Some researchers believe that CO_2_ is converted to methane by the formation of carbonates, formates, etc. (Aldana et al., 2013; Ilsemann et al., 2019). Wang W. et al. (2016) studied the mechanism of CO_2_ methanation over Ni-Ce/CNT catalyst through the diffuse reflaxions infrared fourier transformations spectroscopy (DRIFTS) analysis. As observed in Figure 23, electrons were provided by the doped ruthenium and carbon nanotubes. With the help of the doped agent, the electrons were transferred to the CO_2_ molecules during the process of its activation. The activated CO_2_ dissociated on the surface of Ni, forming carbonate intermediate species. Finally, the atomic hydrogen reacted with the carbonate intermediate to form methane. Takano et al. (2016) also found that many types of carbonates on the Y-doped Ni/ZrO_2_ catalyst were observed and the carbonate was the main adsorbate in the reaction during the process of the DRIFTS analysis. Thus, carbonates were the reaction intermediate for the formation of CH_4_ on the Ni/ZrO_2_ catalyst. They found that the bidentate carbonates were probably the most important intermediates adsorbed on these highly active Y doped catalysts. The combination of Y^3+^ and Ni^2+^ with ZrO_2_ created oxygen vacancies in the oxide, which would help to form a stable bidentate carbonate adsorbate on the catalyst.

**Figure 24:**
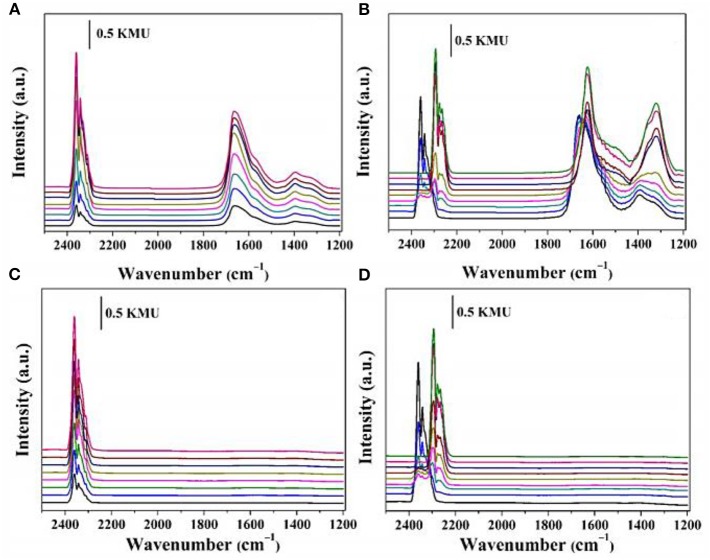
DRIFT spectra recorded at 170°C during 60 min with ^12^CO_2_ as reaction gas and subsequent 60 min reaction by introducing ^13^CO_2_ over: **(A,B)** Ni/MgAl-MMO and **(C,D)** Ni/CNT. From bottom to top: **(A,C)** 0.5, 1.5, 3, 5, 10, 20, 40, 60 min; **(B,D)** 0, 0.5, 1.5, 3, 5, 10, 20, 40, 60 min. Reproduced from Liu J. et al. (2016) with permission from Royal Society of Chemistry.

It was reported that different supports also could cause different reaction intermediates during the CO_2_ methanation processes (Solis-Garcia and Fierro-Gonzalez, 2019). Muroyama et al. (2016) compared the CO_2_ methanation reaction over Ni-based catalysts supported on different metal oxides. Although almost no CO_2_ methanation reaction occurred over Ni/La_2_O_3_ catalyst at 250°C, the chemisorption of CO_2_ by Ni/La_2_O_3_ was more obvious than other samples. In contrast, the strong chemisorption of CO_2_ was not observed over the Ni/Y_2_O_3_ and Ni/Sm_2_O_3_ catalysts. For Ni/Y_2_O_3_ catalysts, the main reaction intermediates of the CO_2_ methanation reaction were carbonate and formate species. The carbonate species was the initial reaction intermediates and then it was gradually converted into monodentate formate and bidentate formate through hydrogenation process. The main reaction pathway for CH_4_ production was hydrogenation of monodentate formate because the reaction rate of monodentate was faster than that of bidentate. CO_2_ methanation process over the Ni/La_2_O_3_ catalyst did not experience bicarbonate and formate intermediates because of the high desorption temperature of CO_2_. CH_4_ was formed by experiencing the formate intermediate rather than the CO pathway over the Ni/ZrO_2_ catalyst. Furthermore, Pan et al. (2014) also found that the methanation reaction mechanisms over Ni/Al_2_O_3_ and Ni/Y_2_O_3_ were similar. As regard the Ni/Al_2_O_3_, CO_2_ reacted directly with surface hydroxyl groups and surface oxygen to form bicarbonate and monodentate carbonate intermediates. The reaction between CO_2_ and the surface hydroxyl group could be attributed to the nucleophilic attack of the oxygen atom of the hydroxyl group on the CO_2_ carbon atom (Solis-Garcia and Fierro-Gonzalez, 2019). Similarly, the bidentate formate appeared at 225°C could be owing to the reaction between the bicarbonate (and/or monodentate) carbonate and H_2_. As the temperature increased, methane characteristic peaks began to form and the intensity of formate slowly decreased. According to previous reports (Guilera et al., 2019), the CO_2_ methanation catalysts commonly possess weak basicity site, medium basicity site, and strong basicity site. The weak basicity sites are usually derived from surface hydroxyl groups and the moderate and strong basicity sites are derived from surface oxygen. Moderate basicity sites promote the decomposition of formate salts. Strong alkaline sites have a negative effect on the activation of carbonates, thereby inhibiting the generation of CH_4_. Therefore, the hydrogenation of monodentate carbonates was hindered by the strong basicity sites of surface oxygen in Ni/Al_2_O_3_. The hydrogenation of bidentate carbonates was the main reaction pathway for CH_4_ formation. The methanation reaction of Ni/CeO_2_-ZrO_2_ was different with that of Ni/Y_2_O_3_. The hydrogen carbonate, bidentate carbonate and monodentate carbonate intermediates appeared over Ni/CeO_2_-ZrO_2_ during the methanation reaction. As the temperature increased, the number of carbonate species gradually decreased and the number of bidendate species gradually increased. This indicated that the bidentate species were main intermediates in the reaction (Pan et al., 2014). According to the report by Wang F. et al. (2016), CO_2_ was mainly converted to ${\text{CO}}_{2}^{\text{δ}-}$ on Ru/CeO_2_ catalyst while CO_2_ was converted to bicarbonate on Ru/α-Al_2_O_3_ catalyst. Besides, Wei et al. (Liu J. et al., 2016) found that the doped additive affected the dominant reaction pathway during the process of CO_2_ methanation. As shown in Figure 24, carbonate and hydrocarbonate were identified immediately over the Mg-assisted Ni catalyst. However, the formation of any relevant intermediates was hardly observed in the case of Ni/carbon nanotube (CNT) catalyst because MgO base sites promoted the activation of CO_2_ molecule to carbonate/hydrocarbonate species and Ni/CNT lacked base sites. Further, CO_2_ was mainly converted into methane via the formate intermediate over the Ni-based catalyst prepared by the ammonia evaporation method. However, the formate intermediate could not be formed over the Ni/CeO_2_-ZrO_2_ catalyst prepared by wet impregnation method (Ashok et al., 2017). In general, the nature of the catalytic support, additive and preparation strategy of the catalyst could affect the dominant reaction pathway and intermediates during the process of the methanation of CO_2_.

As for the activation site of CO_2_, some scholars believe that the activation of CO_2_ occurs over the metallic Ni active site (Aziz et al., 2014b). Solis-Garcia and Fierro-Gonzalez (2019) reported the mechanistic path of CO_2_ methanation on Ni/MSN. Specifically, CO_2_ and H_2_ molecules were firstlyadsorbed on Ni active sites, followed by dissociation to form CO, O, and H atoms, and migrated onto the MSN surface. The CO then interacted with oxide surfaces of the MSN and bidentate formate was also formed through the interaction with atomic hydrogen. Meanwhile, the O atom spilt over onto the surface of the MSN and was stabilized in the oxygen vacancy site near the Ni sites. However, the viewpoint of Jangam et al. (2019) was opposite. They found that carbonate was generated by the reaction of CO_2_ with a surface basic oxyanion on the metal oxide. Therefore, the activation of CO_2_ occurred on the support. Besides, Aldana et al. (2013) also found that CO_2_ was activated on the CeO_2_-ZrO_2_. Therefore, up to now, the real activation site of the CO_2_ molecule has still been the controversy of the CO_2_ methanation reaction.

## Conclusions and Perspective

Nowadays, CO_2_ emissions are increasing, resulting in a series of global environmental problems. Therefore, it is of greatly practical significance to study CO_2_ methanation technology in order to solve the problem of energy shortage and reduce the concentration of CO_2_ in the atmosphere. Besides, it is of great urgency to develop highly efficient Ni based catalyst to accelerate this process because of the kinetic limitations of CO_2_ methanation.

In recent years, Ni-based catalysts have been widely used due to their good catalytic performance and low price. In order to develop Ni-based catalysts with good low-temperature activity, the global scientists have made great efforts to investigate different influencing factors, such as catalytic support, dopant, preparation method. This review generally summarizes different ways to improve catalyst performance. From the perspective of preparation methods, the traditional preparation methods of Ni-based catalysts, such as impregnation and coprecipitation, have become basically mature technologies. Therefore, the researchers have begun to develop new methods and strategies, such as microwave assisted method and plasma method. The catalytic performance of the catalyst prepared by these methods is usually superior to that by the conventional preparation method. In the viewpoint of dopants, their addition can adjust the acidity-alkalinity and electronic/redox property of the catalyst, further improving the dispersion and coordination environment of the metallic active sites. As a result, the additive-modified Ni-based catalyst has the advantages of good reactivity and long lifespan. In this review, the additives are divided into four categories, namely rare earth metals, alkaline earth metals, transition metals and precious metals, and their effects on the catalyst are described and summarized in detail. As for the catalytic supports, they can obviously influence the textural property of the catalyst, thereby affecting the catalytic performances toward CO_2_ methanation process. In terms of reaction conditions, such as temperature, pressure, humidity, etc., they are also considered as important factors affecting the catalytic behavior of the catalyst, which have been carefully summarized in this review. In addition, the reaction mechanism of Ni based catalysts toward CO_2_ methanation are also carefully reviewed. It was believed that the nature of the catalytic support, additive and preparation strategy of the catalyst could affect the dominant reaction pathway and intermediates during the process of the methanation of CO_2_.

In future research, the hot spots of research on Ni-based catalysts toward CO_2_ methanation should still be placed on the improvement of both low-temperature catalytic performance and anti-sintering property of the metallic Ni active site. The development of new supports ought to be the main research line. Besides, the investigation of CO_2_ methanation mechanism on the Ni-based catalyst is beneficial to finding routes to improve the activity of catalysts. Meanwhile, it is of great significance to optimize the preparation method in order to scale up the synthesis of the catalysts in the future industrialization process. Finally, taking the cost of preparing catalysts into consideration is necessary in order to achieve green catalysis and catalysts should be applied to industrial CO_2_ methanation by improving reactors and other methods.

## Author Contributions

MC, LX, and CL: conceptualization. YC and XH: software. MC, LX, and CW: validation. XW, YL, and BY: investigation. MC, XH, and QS: resources. ZM, BY, and CW: data curation. CL: writing—original draft preparation. CL, LX, and MC: writing—review and editing. MC and LX: supervision, methodology, and project administration.

## Conflict of Interest

The authors declare that the research was conducted in the absence of any commercial or financial relationships that could be construed as a potential conflict of interest.
